# Branched-chain amino acid transaminase 1 confers EGFR-TKI resistance through epigenetic glycolytic activation

**DOI:** 10.1038/s41392-024-01928-8

**Published:** 2024-08-15

**Authors:** Tao Zhang, Zilu Pan, Jing Gao, Qingqing Wu, Gang Bai, Yan Li, Linjiang Tong, Fang Feng, Mengzhen Lai, Yingqiang Liu, Peiran Song, Yi Ning, Haotian Tang, Wen Luo, Yi Chen, Yan Fang, Hui Zhang, Qiupei Liu, Yudi Zhang, Hua Wang, Zhiwei Chen, Yi Chen, Meiyu Geng, Hongbin Ji, Guilong Zhao, Hu Zhou, Jian Ding, Hua Xie

**Affiliations:** 1 Division of Antitumor Pharmacology & Analytical Research Center for Organic and Biological Molecules & State Key Laboratory of Drug Research & Small-Molecule Drug Research Center, Shanghai Institute of Materia Medica, Chinese Academy of Sciences, Shanghai, China; 2 University of Chinese Academy of Sciences, Beijing, China; 3 Zhongshan Institute for Drug Discovery, Shanghai Institute of Materia Medica, Chinese Academy of Sciences, Zhongshan, China; 4 School of Pharmaceutical Sciences, Southern Medical University, Guangzhou, China; 5 Shanghai Lung Cancer Center, Shanghai Chest Hospital, Shanghai Jiao Tong University School of Medicine, Shanghai, China; 6 Department of Chemical and Environmental Engineering, University of Nottingham, Ningbo, China; 7 School of Life Science and Technology, ShanghaiTech University, Shanghai, China; 8 State Key Laboratory of Cell Biology, Shanghai Institute of Biochemistry and Cell Biology, Center for Excellence in Molecular Cell Science, Chinese Academy of Sciences, Shanghai, China

**Keywords:** Cancer metabolism, Lung cancer

## Abstract

Third-generation EGFR tyrosine kinase inhibitors (TKIs), exemplified by osimertinib, have demonstrated promising clinical efficacy in the treatment of non-small cell lung cancer (NSCLC). Our previous work has identified ASK120067 as a novel third-generation EGFR TKI with remarkable antitumor effects that has undergone New Drug Application (NDA) submission in China. Despite substantial progress, acquired resistance to EGFR-TKIs remains a significant challenge, impeding the long-term effectiveness of therapeutic approaches. In this study, we conducted a comprehensive investigation utilizing high-throughput proteomics analysis on established TKI-resistant tumor models, and found a notable upregulation of branched-chain amino acid transaminase 1 (BCAT1) expression in both osimertinib- and ASK120067-resistant tumors compared with the parental TKI-sensitive NSCLC tumors. Genetic depletion or pharmacological inhibition of BCAT1 impaired the growth of resistant cells and partially re-sensitized tumor cells to EGFR TKIs. Mechanistically, upregulated BCAT1 in resistant cells reprogrammed branched-chain amino acid (BCAA) metabolism and promoted alpha ketoglutarate (α-KG)-dependent demethylation of lysine 27 on histone H3 (H3K27) and subsequent transcriptional derepression of glycolysis-related genes, thereby enhancing glycolysis and promoting tumor progression. Moreover, we identified WQQ-345 as a novel BCAT1 inhibitor exhibiting antitumor activity both in vitro and in vivo against TKI-resistant lung cancer with high BCAT1 expression. In summary, our study highlighted the crucial role of BCAT1 in mediating resistance to third-generation EGFR-TKIs through epigenetic activation of glycolysis in NSCLC, thereby supporting BCAT1 as a promising therapeutic target for the treatment of TKI-resistant NSCLC.

## Introduction

Resistance to epidermal growth factor receptor (EGFR) tyrosine kinase inhibitors (TKIs) has been considered the primary cause of treatment failure and cancer recurrence in patients with EGFR-mutant non-small cell lung cancer (NSCLC). Among multiple resistance mechanisms, the secondary mutation T790M in EGFR exon 20 is recognized as the most prevalent cause of acquired resistance to first- and second-generation EGFR TKIs.^1^ Several third-generation EGFR TKIs are designed to irreversibly target EGFR with the T790M resistance mutation as well as EGFR activating mutations. Among them, osimertinib (AZD9291) stands out as a representative compound widely approved for the standard second-line therapy of NSCLC with EGFR T790M mutation and first-line treatment in NSCLC with EGFR activating mutations.^2^ Additionally, ASK120067 is a novel third-generation EGFR TKI with promising antitumor efficacy reported by our team and is currently undergoing the New Drug Application (NDA) submission in China.^3^ Despite the marked preclinical and clinical efficacy of third-generation EGFR TKIs, acquired resistance inevitably develops. Various mechanisms have been reported, including EGFR mutations disrupting drug binding (e.g., C797S), activation of alternate pathways, aberrant downstream signaling and lineage plasticity leading to small cell transformation.^4,5^ However, over 30% of resistance mechanisms to osimertinib remain unexplained, which attracts the exploration of new resistance mechanisms and potential therapeutic strategies for the treatment of TKI-resistant NSCLC.^6^

Metabolic reprogramming is considered one of the main hallmarks of malignant tumors and has profound impacts on genome stability, cell proliferation and the tumor microenvironment.^7^ As fundamental bricks of cell structure, amino acids provide building blocks for protein synthesis and serve as sources of energy and metabolites required by proliferating cells.^8^ Branched-chain amino acids (BCAAs), including valine, leucine and isoleucine, are essential amino acids that not only serve as nutrient substrates, but can also be degraded to provide multiple metabolites for other pathways involved in oncogenesis.^9,10^ In cells, branched-chain amino acid transaminases (BCATs), including cytosolic BCAT1 and mitochondrial BCAT2, initiate BCAA metabolism by reversibly transferring the amino group from BCAAs to alpha-ketoglutarate (α-KG) to produce glutamate and their corresponding branched-chain keto acids (BCKAs). Subsequently, the BCKAs undergo decarboxylation by the branched-chain α-keto acid dehydrogenase (BCKDH) complex in the mitochondria and eventually metabolized into acetyl-coenzyme A (acetyl-CoA) and succinyl-CoA to fuel the tricarboxylic acid (TCA) cycle.^11^ Altered BCAA metabolism is associated with tumor progression in many types of human cancers, including glioblastoma, pancreatic ductal adenocarcinoma, breast cancer, leukemia, hepatocellular carcinoma and NSCLC.^12–14^ Mayers et al. revealed that *Kras*-driven NSCLC incorporated free BCAAs as nitrogen resources for nucleotide synthesis to support tumor growth.^12^ More strikingly, upregulated BCAT1 and reprogrammed BCAA metabolism in NSCLC cells have been reported to attenuate reactive oxygen species (ROS) accumulation and mediate short-term tolerance to first-generation EGFR TKIs.^15^ While these studies present the potential oncogenic roles of BCAA metabolism in NSCLC, how tumor cells orchestrate oncogenic BCAA metabolism to support NSCLC progression remains largely elusive. Moreover, no agents targeting BCAA metabolism have been reported for NSCLC treatment.

α-KG plays a critical role in multiple biological processes, including metabolic reprogramming, signaling modules and genetic regulation.^16^ As the substrate of 2-oxoglutarate (2-OG)-dependent dioxygenases, α-KG stimulates hydroxylases such as prolyl hydroxylases (PHDs) and factor inhibiting HIF-1α 1 (FIH-1) to restrict the activity of HIF-1α signaling. Moreover, α-KG impacts gene expression and cellular stemness by activating the Jumonji-C (JmjC) family of lysine demethylases (KDMs) (JmjC-KDMs) and ten-eleven translocation (TET) enzymes which mediate histone- and DNA demethylation, respectively.^17^ Additionally, glutamate dehydrogenase 1 (GLUD1) mediated α-KG production contributes to triggering epithelial-mesenchymal transition in docetaxel- or gefitinib-resistant lung cancer.^18^ Nonetheless, the precise mechanisms linking α-KG to drug resistance remain unclear.

In this study, we employed high-throughput proteomics analysis to characterize proteome alterations upon acquired resistance, aiming to identifying new resistance mechanisms to third-generation EGFR TKIs. BCAT1 emerged as a highly upregulated protein in TKI-resistant cells compared to TKI-sensitive cells and plays a key role in supporting cell survival and maintaining drug resistance in vitro and in vivo. Mechanistically, overexpression of BCAT1 accelerated BCAA anabolism to facilitate α-KG-dependent epigenetic activation of glycolysis genes in TKI-resistant cells, consequently enhancing glycolysis to maintain cellular viability and mediate drug resistance. Moreover, we developed and characterized WQQ-345 as a novel BCAT1-targeted inhibitor, and demonstrated its in vitro and in vivo antitumor efficacy against TKI-resistant NSCLC tumors.

## Results

### BCAT1 is upregulated in EGFR TKI-resistant tumors and associated with poor prognosis in lung adenocarcinoma

**Fig. 1:**
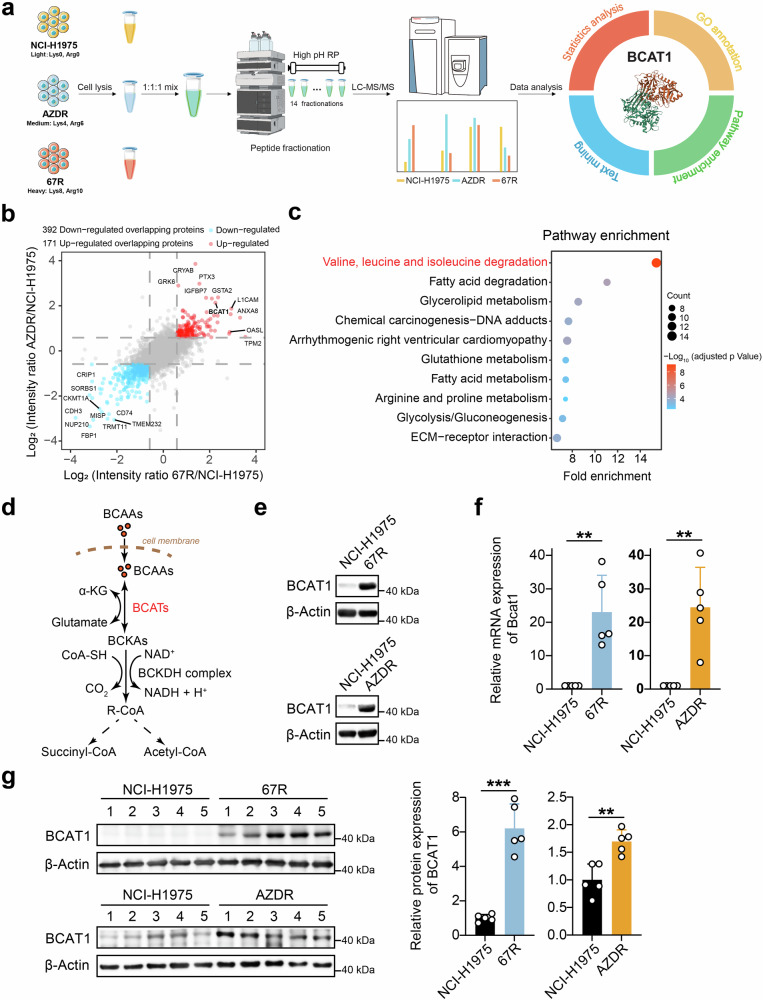
Discovery and validation of enhanced expression of BCAT1 in TKI-resistant lung cancer. **a** Workflow of the SILAC assay to identify differentially expressed proteins between third-generation EGFR TKI-resistant clones and parental cells. Ribbon representation of the experimental structure of BCAT1 (PDB ID 7NTR^42^) is shown on the right. **b** Common differentially expressed proteins in both ASK120067-resistant strains (67R) and osimertinib-resistant strains (AZDR). Red dots: up-regulated overlapping proteins; blue dots: down-regulated overlapping proteins. **c** Pathway enrichment analysis of differentially expressed overlapping proteins in TKI-resistant cells compared to the parental cells. The top 10 enriched pathways in TKI-resistant cells versus parental NCI-H1975 cells are shown. **d** Diagram of BCAT**-**catalyzed reversible BCAAs metabolism. **e**, **f** BCAT1 protein levels and relative mRNA levels (*n* = 5) in the indicated tumor cells were determined by Western blot assay (**e**) and RT‒qPCR (**f**). **g** BCAT1 expression levels in the indicated tumor tissues were determined by Western blot assay (*n* = 5) and are shown as representative images (left) and a quantitative graph (right). *p < 0.05, **p < 0.01, ***p < 0.001. Data are ex*p*ressed as the mean ± SD

EGFR TKI-resistant NSCLC cell lines were generated as previously described by dose escalation exposure of TKI-sensitive NCI-H1975 (EGFR^L858R/T790M^) NSCLC cells to ASK120067 or osimertinib (denoted by 67R and AZDR, respectively).^3^ We further validated the decreased sensitivity of resistant cells to the respective EGFR TKI compared to parental cells using colony formation and cell proliferation assays (Supplementary Fig. 1a, b). To investigate deregulated proteins associated with drug resistance, we conducted stable isotope labeling by amino acids in cell culture (SILAC) assay-based proteomic profiling comparing 67R and AZDR single-cell clones alongside their parental NCI-H1975 cells (Fig. 1a). As shown in Supplementary Fig. 1c, 67R exhibited similar protein expression patterns from that of AZDR in comparison to NCI-H1975 cells, indicating shared mechanisms of drug resistance to third-generation EGFR-TKI agents. To discover potential common resistance biomarker, we extracted the proteins that were significantly differentially expressed in both 67R and AZDR cells, resulting in 171 up-regulated and 392 down-regulated overlapping proteins (Fig. 1b). Pathway enrichment analysis of these overlapping proteins revealed significant dysregulation of metabolic pathways, including BCAA degradation, fatty acid degradation, glycerolipid metabolism, glutathione metabolism, arginine/proline metabolism and glycolysis/gluconeogenesis (Fig. 1c). Remarkably, proteins involved in BCAA degradation was most significantly altered in resistant cells, suggesting potential involvement of reprogrammed BCAA metabolism in EGFR TKI-mediated drug resistance.

Among all the enzymes involved in BCAAs degradation (Fig. 1d), we observed that branched-chain amino acid transaminase 1 (BCAT1) was also a top significantly up-regulated overlapping protein marked in Fig. 1b. As the cytosolic enzyme initiating BCAA metabolism, the increase in BCAT1 expression in the resistant cells was further confirmed at both the mRNA and protein levels (Fig. 1e, f). Moreover, 67R- and AZDR-resistant xenograft tumors also showed elevated expression of BCAT1 compared with the parental tumor tissues (Fig. 1g). Additionally, we observed increased BCAT1 expression under short-term high concentration of ASK120067 treatment in NCI-H1975 cells (Supplementary Fig. 2a). Similar trends were observed in other TKI-sensitive cell models such as PC9 (EGFR^19del^) cells and constructed BaF3 cells expressing EGFR Exon 19del/T790M (BaF3^19del/T790M^) (Supplementary Fig. 2b, c). These results showed the upregulated expression of BCAT1 in both TKI-resistant cells and TKI-tolerant cells. Interestingly, we did not observe markedly upregulated expression of mitochondrial BCAT2 in our resistant cells (Supplementary Fig. 3a, b), suggesting that BCAT1 was the main BCAT enzyme isoform involved in TKI-resistant NSCLC cells.

**Fig. 2:**
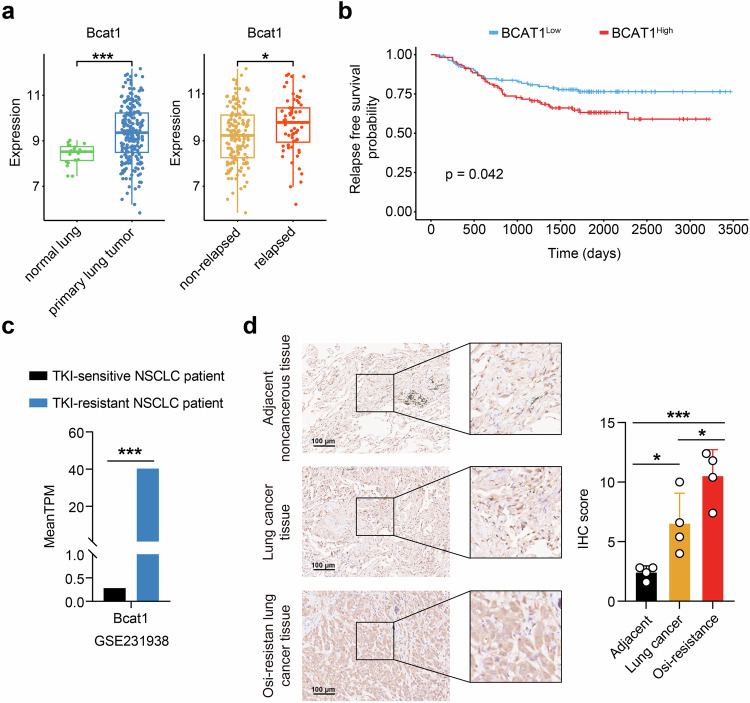
Clinical implications of BCAT1 expression in lung cancer. **a** Comparison of BCAT1 gene expression in normal lung tissue and primary lung adenocarcinoma tumors (left) or in tumor tissues from non-relapsed- and relapsed lung cancer patients (right) in the GSE31210 dataset, which contains 226 patients with primary stage I-II lung adenocarcinomas and 20 normal lung tissues. **b** Relapse-free survival probability of two groups of lung cancer patients classified by BCAT1 median expression levels in the GSE31210 dataset. **c** Comparison of BCAT1 gene expression in tumors from TKI-sensitive- and TKI-resistant NSCLC patients in the GSE231938 dataset. **d** IHC staining of BCAT1 in lung cancer tissues, adjacent noncancerous tissues and osimertinib-resistant tumors (*n* = 4). Results are shown as representative images and quantitative graphs. *p < 0.05, ***p < 0.001. Data are expressed as the mean ± SD

To assess the clinical relevance of BCAT1 in lung cancer, we analyzed specimens from a cohort of patients with primary lung adenocarcinoma (LUAD) and samples of normal lung tissues using the GSE31210 dataset from the Gene Expression Omnibus (GEO) database. Patients with primary LUAD were further categorized into relapse and non-relapse subgroups based on the observations during the follow-up period. Remarkably, BCAT1 exhibited significant overexpression in primary LUAD tumors compared with normal lung tissues. Furthermore, its expression was higher in tumors from relapsed LUAD patients than in those from the non-relapsed group (Fig. 2a). Additionally, BCAT1 expression showed an inverse correlation with relapse-free survival (RFS) time in LUAD patients (Fig. 2b). Moreover, analysis of the supplemental database (GSE231938) revealed elevated BCAT1 expression in samples from TKI-resistant NSCLC patients compared to those from TKI-sensitive NSCLC patients (Fig. 2c). These results indicated that BCAT1 may serve as a potential biomarker associated with poor prognosis and TKI resistance in lung cancer. In contrast, although the expression of BCAT2 was upregulated in primary LUAD tumors in comparison to normal lung tissues, there was no difference in BCAT2 expression between relapsed tumors and non-relapsed group (Supplementary Fig. 3c), or between TKI-sensitive and TKI-resistant patients (Supplementary Fig. 3e). Additionally, no significant correlation was observed between BCAT2 expression and tumor relapse in LUAD patients (Supplementary Fig. 3d).

Besides clinical database analysis, we collected clinical tumor samples and detected BCAT1 expression using immunohistochemical staining. As illustrated in Fig. 2d, BCAT1 expression levels were markedly elevated in lung cancer tumors compared with adjacent noncancerous tissues. Furthermore, we observed an even greater elevation of BCAT1 levels in osimertinib-resistant tumors compared to lung cancer tumors. These findings underscore the clinical relevance of BCAT1 in lung cancer, particularly in TKI-resistant cases.

### BCAT1 confers EGFR TKI resistance in vitro and in vivo

**Fig. 3:**
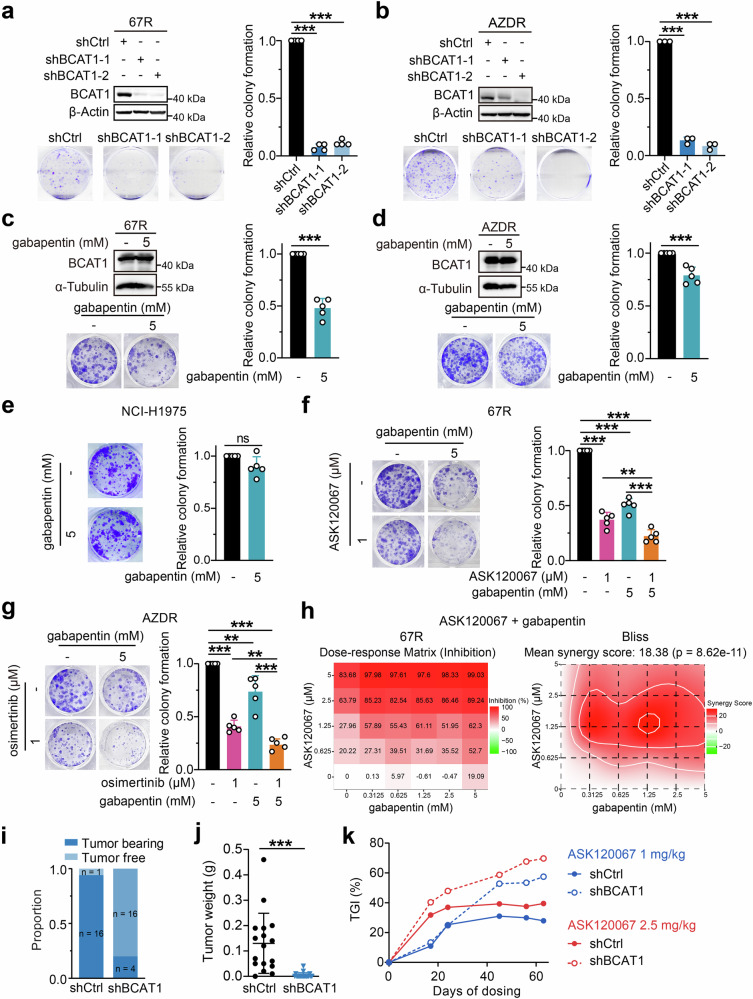
BCAT1 sustained cell survival and conferred drug resistance to third-generation EGFR TKIs. **a**, **b** Colony formation of 67R (*n* = 4) (**a**) or AZDR (*n* = 3) (**b**) cells with or without BCAT1 knockdown was measured. **c**, **d**, **e** The anti-growth effects of gabapentin in 67R (**c**), AZDR (**d**) and NCI-H1975 (**e**) cells were assessed by colony formation assay (*n* = 5). **f**, **g** Effects of combination therapy on cellular growth in 67R (**f**) or AZDR (**g**) cells were evaluated. All the above colony formation results are shown as representative images and quantitative graphs (*n* = 5). **h** Effects of gabapentin in combination with EGFR TKI in 67R cells were assessed using Sulforhodamine B assay and showed as dose-response matrix (left) and synergy score matrix (right). **i**, **j** In vivo tumor growth of control 67R (shCtrl) tumors and BCAT1-knockdown 67R (shBCAT1, same as shBCAT1-1) tumors. Equal numbers of cells (10^7^ cells/mouse) were subcutaneously injected into the right flank of BALB/c nude mice. Mice were sacrificed 58 days after grafting, and the tumor formation proportion (**i**), tumor weight (**j**) were evaluated or showed (*n* = 17 for shCtrl group, *n* = 20 for shBCAT1 group). **k** Tumor growth inhibition (TGI) effects of ASK120067 (oral administration, 1 mg/kg or 2.5 mg/kg, once daily) for 63 days in control 67R (shCtrl) and BCAT1-knockdown 67R (shBCAT1) tumor models were evaluated. **p < 0.01, ***p < 0.001; ns, not significant. Data are expressed as the mean ± SD

Given BCAT1’s upregulation in EGFR TKI-resistant cells in vitro and in vivo, and its clinical implications, we assessed the effects of BCAT1 on resistant cell growth and survival using a colony formation assay. Notably, knockdown of BCAT1 by specific short hairpin RNA (shRNA) markedly suppressed colony formation in both 67R and AZDR cells (Fig. 3a, b). Treatment with the BCAT1 inhibitor gabapentin selectively decreased resistant cell formation (Fig. 3c, d) without affecting the growth of parental NCI-H1975 cells (Fig. 3e). Moreover, pharmacological inhibition of BCAT1 by gabapentin sensitized 67R and AZDR cells to ASK120067 and osimertinib, respectively (Fig. 3f, g). Similar results were observed using a cell proliferation assay, where gabapentin selectively suppressed cell proliferation in 67R and AZDR cells compared with NCI-H1975 cells (Supplementary Fig. 4a) and combination of gabapentin and ASK120067 or osimertinib exhibited synergistical anti-growth effects in the corresponding resistant cells (Fig. 3h and Supplementary Fig. 4b). These results demonstrated that BCAT1 was required for cell survival and and drug resistance in TKI-resistant cells. While no significant changes were observed in BCAT1 protein levels upon gabapentin or EGFR TKI treatment (Fig. 3c, d and Supplementary Fig. 4e), consistent with the reported target selectivity of EGFR TKIs and gabapentin as an inhibitor of BCAT1 enzyme activity, we surprisingly found that the combination of gabapentin and EGFR TKI synergistically decreased BCAT1 protein levels in TKI-resistant cells (Supplementary Fig. 4e), a phenomenon not previously reported. We speculate that this could be due to the selection of non-responding lower-BCAT1 67R populations or to complicated signaling crosstalk, which still needs further elucidation.

To further determine the functional role of BCAT1 in a mouse model, we established subcutaneous xenograft models using 67R cells stably expressing either shCtrl or shBCAT1. We found that knockdown of BCAT1 significantly impaired the ability of 67R cells to form tumors, leading to a noticeable decrease in both tumor formation rate and tumor weight (Fig. 3i, j and Supplementary Fig. 4f). Additionally, in the context of long-term treatment, ASK120067 exhibited enhanced antitumor efficacy against BCAT1-knockdown 67R tumors compared to control 67R tumors, as evidenced by tumor growth inhibition rates (TGI) of 57.46% versus 27.87% at a dose of 1 mg/kg and 69.70% versus 39.46% at a dose 2.5 mg/kg on day 63 (Fig. 3k and Supplementary Fig. 4g, h). These findings confirmed the critical role of BCAT1 in the survival of resistant NSCLC cells and its mediation of drug resistance to third-generation EGFR TKIs in vitro and in vivo.

### BCAT1 facilitates α-KG generation to support cell growth of resistant cells

**Fig. 4:**
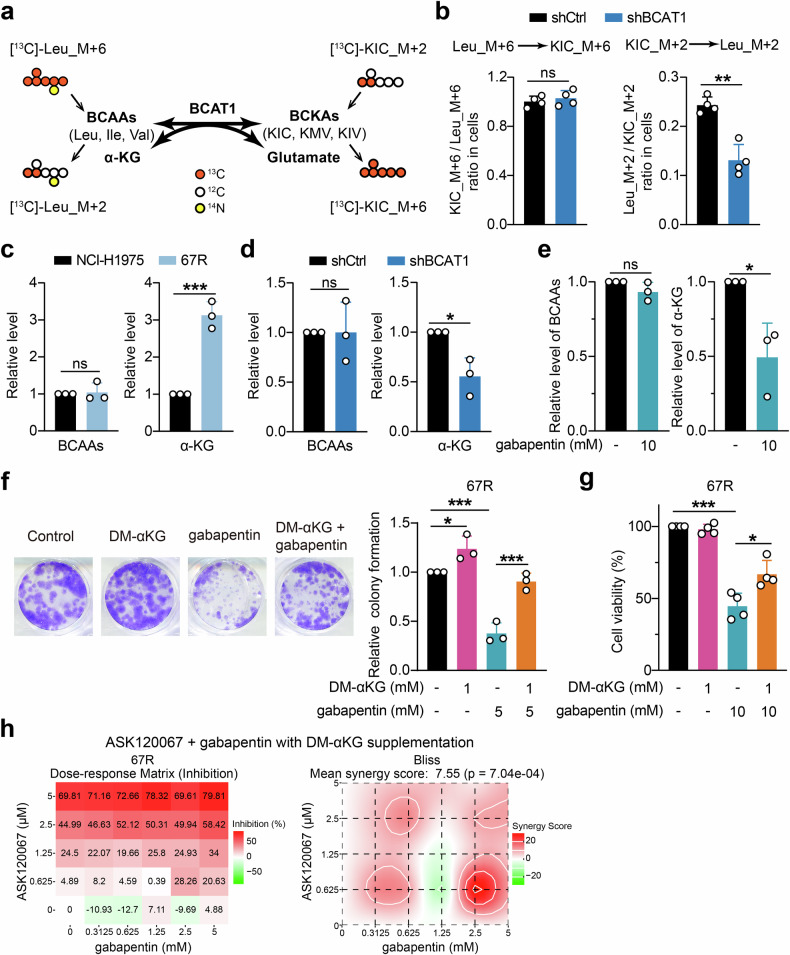
Increased α-KG production was involved in BCAT1-mediated cell growth and resistance. **a** Schematic outline of BCAT1-mediated reversible transamination and isotope tracing experiment. **b** Ratios of the labeled metabolites in control 67R cells (shCtrl) and BCAT1-knockdown 67R cells (shBCAT1) (*n* = 4). **c** Comparison of intracellular BCAA levels and α-KG levels in NCI-H1975 and 67R cells (*n* = 3). **d, e** Effects of BCAT1 knockdown (**d**) or the BCAT1 inhibitor gabapentin (**e**) on cellular BCAA levels and αKG levels in 67R cells (*n* = 3). **f** Colony formation of 67R cells upon dimethyl-KG (DM-αKG), gabapentin or combination treatment was evaluated and shown as representative images and a quantitative graph (*n* = 3). **g** Cell viability of 67R cells upon dimethyl-KG (DM-αKG), gabapentin or combination treatment was evaluated using SRB colorimetric assay (*n* = 4). **h** Anti-proliferation effects of gabapentin and ASK120067 with DM-αKG supplementation on 67R cells were detected using SRB colorimetric assay, and showed as dose-response matrix (left) and synergy score matrix (right). Synergy scores were calculated by SynergyFinder using Bliss model. *p < 0.05, **p < 0.01, ***p < 0.001; ns, not significant. Data are expressed as the mean ± SD

As BCAT1 was a key enzyme responsible for the reversible conversion from BCAAs and α-KG into BCKAs and glutamate, we next sought to explore how elevated BCAT1 reprogrammed BCAA metabolism in resistant cells using stable isotope tracing. 67R cells stably expressing shCtrl or shBCAT1 were incubated with [^13^C]-Leu_M + 6 and [^13^C]-KIC_M + 2 and analyzed for their specific resulting metabolites [^13^C]-KIC_M + 6 and [^13^C]-Leu_M + 2, respectively (Fig. 4a). We found that BCAT1 knockdown significantly decreased the cellular ratio of [^13^C]-Leu_M + 2/[^13^C]-KIC_M + 2 without markedly affecting the ratio of [^13^C]-KIC_M + 6/ [^13^C]-Leu_M + 6. This indicated that highly expressed BCAT1 in resistant cells might accelerate BCAA anabolism more than BCAA degradation (Fig. 4b).

We further confirmed the changes in BCAA anabolic metabolites in resistant cells and found that intracellular α-KG accumulated significantly in 67R cells compared to parental NCI-H1975 cells, while the total free BCAA pool remained unchanged (Fig. 4c). Additionally, genetic or pharmacological inhibition of BCAT1 resulted in a significant decrease in intracellular α-KG levels in 67R cells, whereas no significant reduction in BCAA levels was observed upon BCAT1 knockdown or inhibition (Fig. 4d, e). These results suggested that the upregulated BCAT1 in resistant cells mainly led to the accumulation of cellular α-KG rather than BCAAs, and we speculated that the sufficient pool of BCAAs from cell-culture media might be the reason for BCAA homeostasis upon changed BCAT1 in tumor cells. We further explored how α-KG affected cell growth in resistant cells. As shown in Fig. 4f, exogenous supplementation with a cell-permeable derivative of α-KG (dimethyl-α-KG, DM-αKG) promoted cell colony formation and significantly rescued the antitumor effect of gabapentin on 67R cells. Moreover, cell proliferation assay showed that supplementation with α-KG not only rescued the growth inhibition caused by gabapentin (Fig. 4g) but also compromised the synergistic anti-growth effects of the gabapentin and ASK120067 combination in 67R cells (Fig. 3h and Fig. 4h). Similar results were observed in AZDR cells (Supplementary Fig. 4b–d). Taken together, our results demonstrated that the increase in the BCAA anabolic product α-KG contributed to BCAT1-mediated cell survival in resistant cells.

### BCAT1 promotes drug resistance through α-KG-dependent H3K27 demethylation

**Fig. 5:**
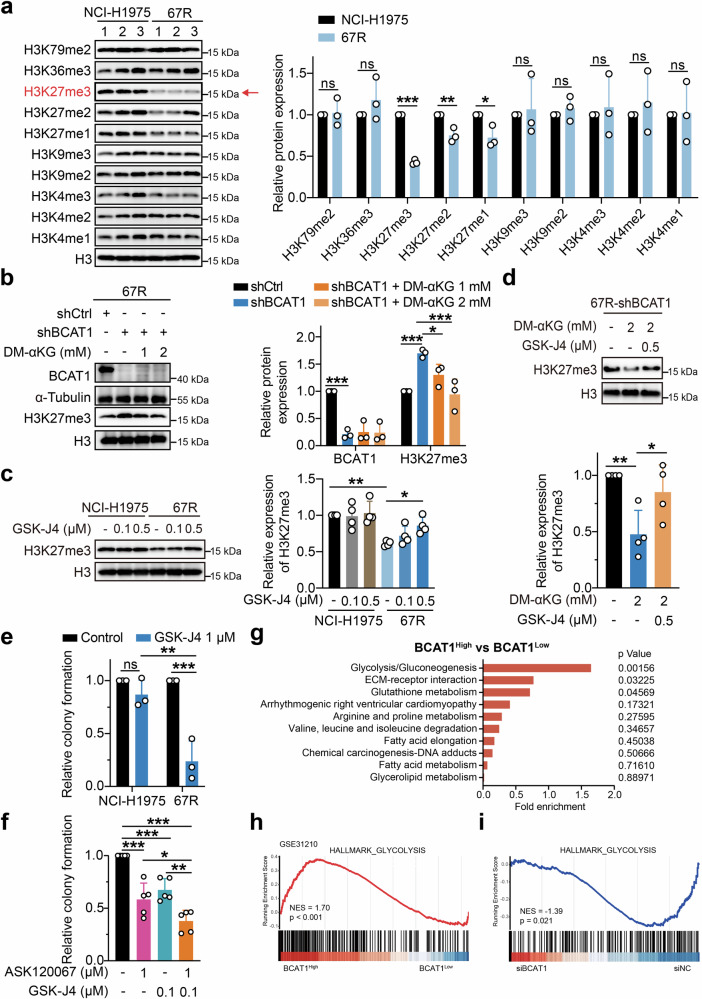
BCAT1 promoted α-KG-dependent H3K27 demethylation in resistant cells. **a** The indicated histone methylation levels in NCI-H1975 cells and 67R cells were determined using Western blotting and are shown as representative images (left) and densitometric quantitative results (right) (*n* = 3). **b** The effects of BCAT1 knockdown and cell-permeable dimethyl-αKG (DM-αKG) supplementation on H3K27me3 expression in 67R cells were detected by Western blotting and are shown as representative images (left) and a quantitative graph (right). Cells were treated with the indicated agent for 24 h (*n* = 3). shCtrl: 67R-shControl; shBCAT1: 67R-shBCAT1. **c** NCI**-**H1975 cells and 67R cells were treated with the indicated doses of GSK-J4 for 48 h, and the protein levels of H3K27me3 were determined by Western blot assay (*n* = 4). Representative images (left) and quantification results (right) are shown. **d** The effects of α-KG and GSK-J4 on H3K27me3 levels in BCAT1-knockdown 67R (shBCAT1) cells were determined. Cells were treated with 2 mM DM-αKG for 24 h and/or 0.5 µM GSK-J4 for 48 h (*n* = 4). Representative immunoblotting images (top) and quantitative graphs (bottom) are shown. **e** The anti-growth effects of GSK-J4 in NCI-H1975 or 67R cells were detected using a colony formation assay (*n* = 3). **f** Relative colony formation of 67R upon ASK120067, GSK-J4, or drug combination treatment (*n* = 5). **g** KEGG pathway enrichment analysis of pathways presented in Fig. 1c for BCAT1-high expression samples versus BCAT1-low expression samples from the clinical dataset GSE31210. The samples of the dataset were stratified based on high versus low expression (cutoff, median) of BCAT1 mRNA in lung adenocarcinoma tumors. **h** Gene set enrichment analysis (GSEA) showed that the glycolysis pathway was enriched in the BCAT1 high expression phenotype of the clinical dataset GSE31210. **i** BCAT1-knockdown 67R (siBCAT1) cells and control 67R (siNC) cells were subjected to transcriptomic analysis, and GSEA demonstrated downregulation of the glycolysis pathway in siBCAT1 cells. *p < 0.05, **p < 0.01, ***p < 0.001; ns, not significant. Data are expressed as the mean ± SD

α-KG is an important metabolite involved in the metabolic control of cell fate by serving as a cofactor for several epigenetic-modifying enzymes, including the Jumonji-C (JmjC) family of lysine demethylases (KDMs) (JmjC-KDMs) and ten-eleven translocation (TET) methylcytosine dioxygenases.^19^ To determine whether the accumulation of α-KG in resistant cells promoted tumor progression via chromatin modulation, we first assessed the histone lysine methylation patterns known to be partly regulated by α-KG-dependent demethylases.^20^ As shown in Fig. 5a, 67R cells exhibited a dramatic decrease in H3K27me3 expression compared to parental NCI-H1975 cells. Moreover, the levels of H3K27me2 and H3K27me1 were also lower in 67R cells, while no significant changes in H3K79me2, H3K36me3, H3K9me1/2/3 and H3K4me1/2/3 were observed between 67R and NCI-H1975 cells.

As H3K27me3 demethylation was reported to promote the transition to a drug-tolerant state during chemotherapy treatment and was most significantly affected in 67R-resistant cells,^21^ we next sought to investigate whether BCAT1 mediated α-KG accumulation led to the downregulated methylation of H3K27me3. Notably, BCAT1 knockdown markedly increased global H3K27me3 expression, which was rescued by supplementation with α-KG (Fig. 5b). This indicates that highly expressed BCAT1 in 67R cells negatively regulated H3K27me3 in an α-KG-dependent manner. We further treated NCI-H1975 and 67R cells with GSK-J4, which is a cell-permeable inhibitor that preferentially suppresses KDM6A and KDM6B, two H3K27me3-specific JmjC-KDMs requiring α-KG as a cofactor,^22^ and investigate the effect of GSK-J4 on H3K27me3 expression and cell growth. As expected, GSK-J4 dose-dependently upregulated H3K27me3 in 67R-resistant cells without affecting the levels of H3K27me3 in NCI-H1975 cells (Fig. 5c). Moreover, GSK-J4 treatment rescued the decrease in H3K27me3 level observed upon α-KG supplementation in BCAT1-knockdown 67R cells (Fig. 5d), suggesting an important role of KDM6A/6B in regulating α-KG-dependent H3K27me3 demethylation. Additionally, GSK-J4 exhibited enhanced growth-inhibition activity in 67R cells compared to NCI-H1975 cells (Fig. 5e), and treatment with GSK-J4 further sensitized 67R cells to ASK120067 inhibition (Fig. 5f).

Additionally, since α-KG also activates TET-dependent DNA demethylation to catalyze 5-methylcytosine (5mC) to 5-hydroxymethylcytosine (5hmC), we also detected the levels of 5mc and 5hmc using a DNA dot blot assay to evaluate TET enzyme activity.^15,17^ As shown in Supplementary Fig. 5a, we did not observe lower 5mC or higher 5hmC levels in 67R cells compared to NCI-H1975 cells, and BCAT1 knockdown hardly affected the levels of 5mC and 5hmC in 67R cells (Supplementary Fig. 5b). Consistently, 67R and NCI-H1975 cells showed similar cell growth inhibition sensitivities to the TET enzyme inhibitor Bobcat339 (Supplementary Fig. 5c). Taken together, the above results indicated that the accumulation of α-KG in TKI-resistant cells mediated by BCAT1 favored the demethylation of the repressive chromatin mark H3K27me3 rather than TET-dependent DNA demethylation.

### Epigenetic regulation of glycolysis-related genes by BCAT1 contributes to drug resistance

Since H3K27me3 was reported as a general marker regulating various biological processes,^23^ we speculated that α-KG-mediated demethylation of H3K27me3 resulted in aberrant transcriptional activation of target genes and subsequent oncogenic signaling to confer drug resistance. To identify the downstream targets regulated by α-KG involved in drug resistance, we reanalyzed the enriched pathways that were significantly changed between 67R and NCI-H1975 cells identified by the SILAC assay (Fig. 1c) in the clinical GSE31210 dataset. We observed that the glycolysis/gluconeogenesis pathway was enriched as the top differential pathway in the BCAT1-high lung adenocarcinoma samples relative to the BCAT1-low tumors (Fig. 5g). Gene set enrichment analysis (GSEA) revealed that high BCAT1 expression was positively correlated with glycolysis gene signatures (Fig. 5h). To further verify the correlation between BCAT1 expression and glycolysis in our resistant cells, we conducted transcriptome-wide RNA sequencing (RNA-seq) and GSEA pathway analysis, and we found that small interfering RNA (siRNA)-mediated BCAT1 knockdown resulted in a significant decrease in the expression of genes in the glycolysis pathway (Fig. 5i and Supplementary Fig. 6a, b), suggesting that glycolysis-related genes are positively regulated by BCAT1 in resistant cells.

**Fig. 6:**
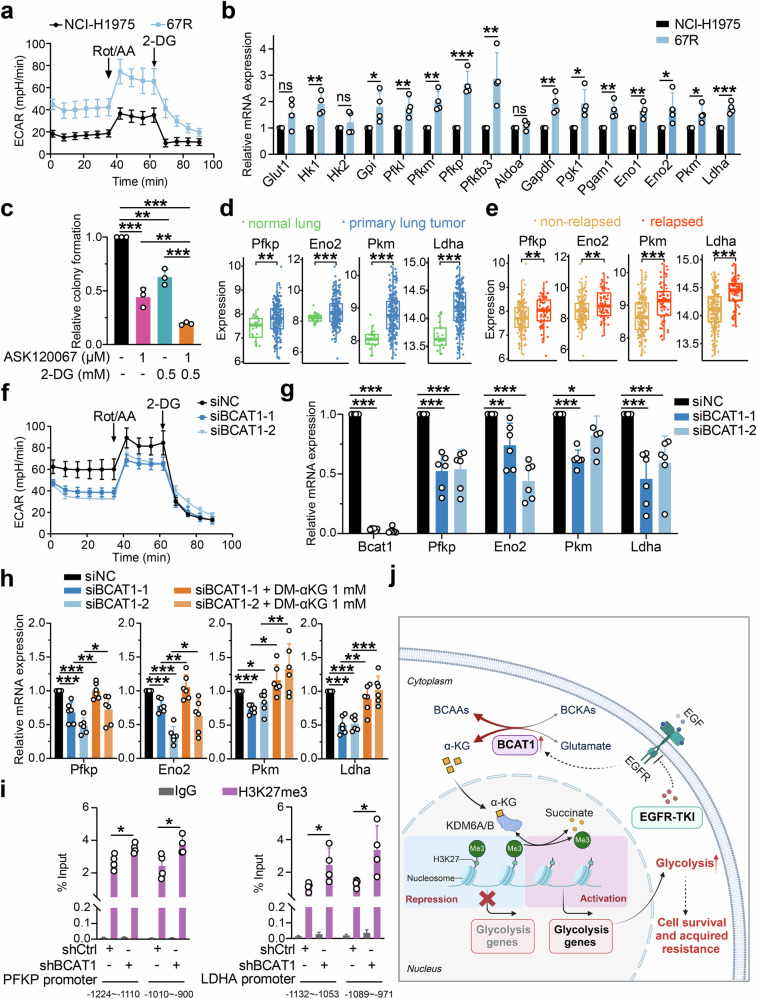
BCAT1 mediated drug resistance by transcriptional activation of glycolysis through α-KG-dependent H3K27 demethylation. **a** Extracellular acidification rates (ECARs) of NCI-H1975 and 67R cells were detected by a Seahorse XF Analyzer. Rot/AA rotenone and antimycin A, 2-DG 2-Deoxy-d-glucose. **b** Relative mRNA levels of glycolytic enzymes in NCI-H1975 and 67R cells were evaluated by RT-qPCR assay (*n* = 4). **c** Quantification of relative colony formation of 67R cells upon ASK120067, 2-DG or combination therapy (*n* = 3). **d**, **e** Comparison of glycolysis genes expression in normal lung tissue and primary lung adenocarcinoma tumors (**d**) or in tumor tissues from non-relapsed and relapsed lung adenocarcinoma patients (**e**) in the GSE31210 dataset. **f** ECAR analysis of control 67R cells (siNC) and BCAT1-knockdown 67R cells (siBCAT1). **g** Relative mRNA levels of the indicated glycolysis-related genes in 67R siNC and 67R siBCAT1 cells were determined by RT-qPCR (*n* = 6). **h** The effects of BCAT1 knockdown and α-KG supplementation on the transcriptional expression of the indicated glycolysis-related genes in 67R. Cells were treated with/without DM-αKG for 24 h before mRNA extraction and RT-qPCR (*n* = 6). **i** ChIP-qPCR analysis of H3K27me3 abundance at the promoters of PFKP (p1, −1224~−1110; p2, −1010~−900) and LDHA (p1, −1132~−1053; p2, −1089~−971) in NC and shBCAT1 67R cells (*n* = 4). **j** Schematic model of BCAT1-mediated EGFR-TKI resistance through α-KG-dependent epigenetic activation of glycolysis. This figure is created with BioRender.com. *p < 0.05, **p < 0.01, ***p < 0.001; ns, not significant. Data are expressed as the mean ± SD

Glycolysis is a predominant metabolic pathway for energy supply and biosynthesis, consisting of more than ten enzymes to transfer glucose to lactate and produce ATP molecules (Supplementary Fig. 6a). Compelling evidence has shown that increasing glycolysis contributes to EGFR TKI resistance and chemoresistance in NSCLC.^24–26^ To explore whether enhanced glucose metabolism was involved in resistance to EGFR TKIs, we first assessed glycolytic activity in TKI-resistant cells and NCI-H1975 cells using a Seahorse XF analyzer. We noticed that both 67R and AZDR exhibited an increased extracellular acidification rate (ECAR), an indicator of intrinsic glycolysis, compared with parental NCI-H1975 cells (Fig. 6a and Supplementary Fig. 6c, 7a). Then, RT-qPCR was performed to detect the transcriptional levels of glycolytic-related genes highlighted in our RNA-seq analysis (Supplementary Fig. 6b). Consistently, a global upregulation of glycolytic-related genes were observed in 67R and AZDR cells compared to NCI-H1975 cells (Fig. 6b and Supplementary Fig. 7b). Importantly, treatment with the glycolysis inhibitor 2-deoxy-D-glucose (2-DG) significantly sensitized 67R and AZDR cells to corresponding TKI treatment (Fig. 6c and Supplementary Fig. 7c). These results suggested that resistant cells exhibited enhanced glycolytic activity to support cell survival under EGFR TKI treatment. Furthermore, analysis of the clinical GSE31210 dataset showed that the transcriptional levels of glycolytic-related ezymes, including PFKP, ENO2, PKM and LDHA, were markedly upregulated in primary LUAD tumors relative to normal lung tissues (Fig. 6d). Moreover, these enzymes showed even higher expression levels in tumors from eventually relapsed LUAD patients compared to those from non-relapsed patients (Fig. 6e), which supported the clinical association of enhanced glycolysis with tumor progression in human lung adenocarcinomas.

We next knocked down BCAT1 in 67R cells using siRNA to validate the impact of BCAT1 on glycolysis. Notably, BCAT1 knockdown impaired glycolytic activity (Fig. 6f) and decreased the transcriptional levels of key glycolytic enzymes, including PFKP, ENO2, PKM and LDHA (Fig. 6g). Similar results were observed in 67R cells upon gabapentin treatment (Supplementary Fig. 6d, e). To determine if BCAT1-induced glucose metabolism reprogramming was dependent on α-KG-mediated epigenetic regulation, we conducted an additional rescue assay with α-KG. As shown in Fig. 6h and Supplementary Fig. 6f, supplementation with α-KG significantly rescued the downregulated mRNA expression of glycolysis genes upon BCAT1 knockdown or inhibition. Furthermore, ChIP-qPCR analysis revealed that BCAT1 knockdown resulted in substantially higher H3K27me3 enrichment on the promoters of PFKP and LDHA (Fig. 6i) in 67R cells, as well as in AZDR cells (Supplementary Fig. 7d). Taken together, these results demonstrated that BCAT1 facilitated BCAA anabolism and α-KG-dependent demethylation of H3K27me3, thereby transcriptionally activating glycolysis genes and contributing to cell survival and drug resistance (Fig. 6j). Strategies targeting these mechanisms, such as pharmacological inhibition of BCAT1, KDM6A/B, and glycolysis, could be effective in reversing EGFR-TKI resistance in NSCLC.

### WQQ-345 is identified as a novel BCAT1 inhibitor with preclinical antitumor efficacy

**Fig. 7:**
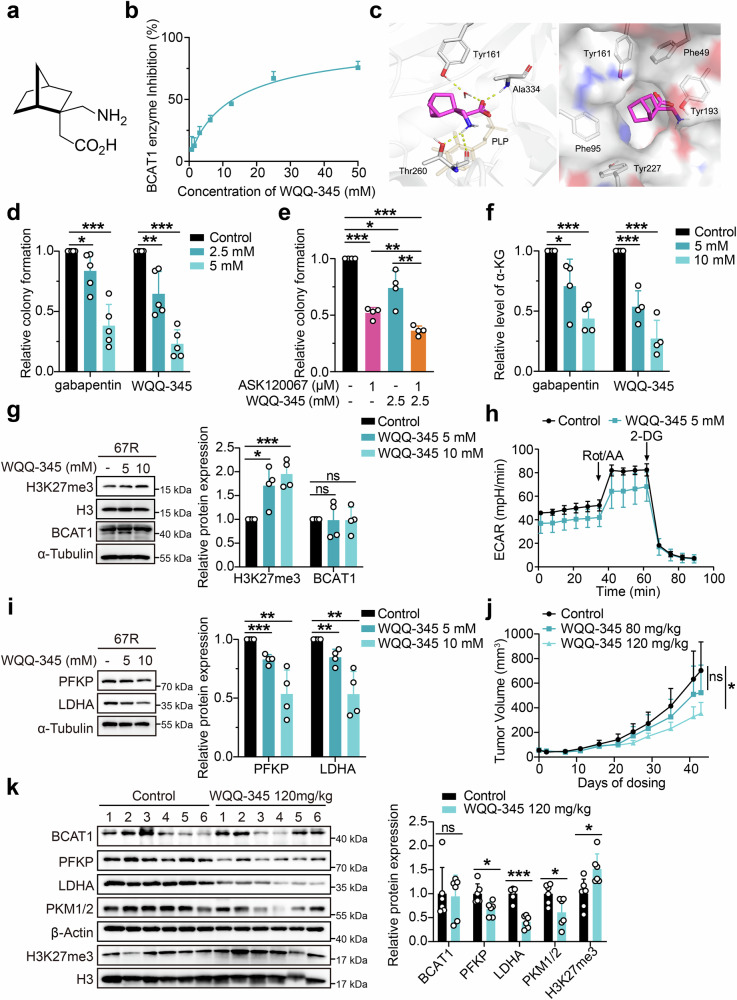
WQQ-345 was identified as a novel BCAT1 inhibitor with antitumor potency. **a** Chemical structure of WQQ-345. **b** Enzyme-inhibition effects of WQQ-345 on recombinant human BCAT1 protein. **c** Binding model of WQQ-345 with BCAT1. Left panel shows WQQ-345 (magenta sticks) bound at the active site of BCAT1. The cofactor PLP was depicted in orange. Right panel shows surface representation of BCAT1 (gray surface) with WQQ-345 (magenta sticks). **d** Colony formation assays were performed to examine the growth of 67R cells treated with gabapentin or WQQ-345 (*n* = 5). **e** Quantification of relative colony formation of 67R cells treated with vehicle control, ASK120067, WQQ-345 and drug combination (*n* = 4). **f** Quantification of α-KG levels in 67R cells treated with vehicle control, gabapentin or WQQ-345 for 3 days (*n* = 4). **g** Western blot analysis of the expression of BCAT1 and H3K27me3 in 67R cells upon WQQ-345 treatment for 3 days. Data are shown as representative images (left) and a quantitative graph (right) (*n* = 4). **h** ECAR analysis of 67R cells with or without WQQ-345 treatment for 3 days. **i** Western blot analysis of the expression of indicated glycolytic enzymes in 67R cells upon WQQ-345 treatment for 3 days. The results are shown as representative images (left) and a quantitative graph (right) (*n* = 4). **j** The in vivo antitumor activity of WQQ-345 was evaluated in a 67R xenograft tumor model. 67R tumor-bearing mice were given oral treatments of PBS control or WQQ-345 twice daily for 43 days, and tumor volume was monitored. **k** The expression of BCAT1, H3K27me3 and indicated glycolysis related enzymes in 67R xenograft tumors at the endpoint of drug treatment was assessed by Western blot assay and shown as representative images (left) and a quantitative graph (right) (*n* = 6). *p < 0.05, **p < 0.01, ***p < 0.001; ns, not significant. Data in (**j**) are expressed as the mean ± SEM, and other data are expressed as the mean ± SD

**Scheme 1:**
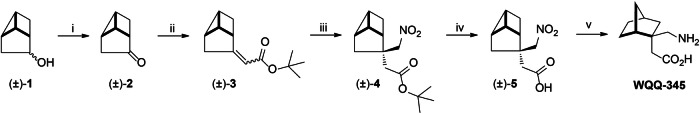
Synthetic route to WQQ-345. Reagents and Conditions: (i) DMSO/(COCl)_2_, (±)−**1**, dried CH_2_Cl_2_, −78 °C, then Et_3_N, −78 °C-rt; (ii) *t*-BuOK, (Et_2_O)_2_P( = O)CH_2_CO_2_Bu^*t*^, dried THF, N_2_, ice-water bath to rt; (iii) DBU, CH_3_NO_2_, 80 °C-85 °C; (iv) aq NaOH, EtOH, reflux; (v) H_2_, Pd(OH)_2_, MeOH, rt

The above findings indicated the potential of targeting BCAT1 as a strategy to overcome EGFR TKI resistance. However, the limited availability of effective BCAT1 inhibitors, with none of which demonstrated significant in vivo antitumor efficacy, highlights the need for further investigation and development of novel BCAT1 inhibitors. Inspired by reports of BCAT1 inhibition by the γ-aminobutyric acid (GABA) derivative gabapentin,^27^ we screened an in-house compound library of GABA derivatives with unique structures, which led to the discovery of WQQ-345 as a BCAT1 inhibitor (Fig. 7a). The synthetic route to WQQ-345 is illustrated in Scheme 1, and detailed synthetic procedures and characterization of the compound against BCAT1 were provided in the supplementary materials and methods. A BCAT1 enzyme activity assay was conducted, and as shown in Fig. 7b, WQQ-345 dose-dependently suppressed the activity of purified recombinant BCAT1 protein, with an IC_50_ of 10.8 mM. To understand the molecular interactions between BCAT1 and WQQ-345, we performed a molecular docking analysis. As detailed in Fig. 7c, WQQ-345 formed direct and water-mediated hydrogen bonds via carboxylate moiety and amino moiety with residues in the BCAT1 active site, including Ala334, Tyr161 and Thr260. At the same time, the norbornane of WQQ-345 formed van der Waals interactions with surrounding residues, such as Phe49, Phe95, Tyr161, Tyr193, and Tyr227. The docking results predicted the binding mode and helped explain the inhibitory potency of WQQ-345 on BCAT1.

Next, we explored the cellular effects of WQQ-345 in our TKI-resistant lung cancer models. A cellular thermal shift assay was conducted to evaluate the thermal stabilization of BCAT1 upon drug binding in 67R cells. Similar to gabapentin, pre-incubation with WQQ-345 improved the thermal stability of cellular BCAT1 at the indicated denaturation temperatures compared to the control, which supported the cellular binding of WQQ-345 to the BCAT1 protein (Supplementary Fig. 8a).

Meanwhile, WQQ-345 dose-dependently reduced the colony formation of 67R resistant cells (Fig. 7d), and its on-target anti-growth effects were further confirmed through a cell proliferation assay, where knockdown of BCAT1 significantly compromised the growth inhibition caused by WQQ-345 in 67R and AZDR cells (Supplementary Fig. 8b, c). Furthermore, co-treatment with WQQ-345 and either ASK120067 or osimertinib exhibited stronger cell growth inhibition than monotherapy (Fig. 7e and Supplementary Fig. 8d), indicating that WQQ-345 could partially restore the sensitivity of resistant cells to the corresponding TKIs. Mechanistically, WQQ-345 treatment led to decreased cellular α-KG levels, upregulated H3K27me3 expression, and resulted in lower expression of glycolytic enzymes (PFKP and LDHA), and impaired glycolysis activity in 67R cells (Fig. 7f–i).

Further animal experiments were conducted to validate the in vivo potency of WQQ-345 in TKI-resistant tumors. As shown in Fig. 7j and Supplementary Fig. 8e, oral administration of WQQ-345 at 120 mg/kg led to robust tumor regression in the 67R subcutaneous xenograft model. Moreover, WQQ-345-treated tumors exhibited increased H3K27me3 expression and decreased levels of PFKP, LDHA, and PKM1/2 compared to the vehicle control tumors (Fig. 7k). Collectively, these results, along with our in vitro observations, confirmed the antitumor and anti-glycolytic effects of WQQ-345, which supported the idea of pharmacological inhibition of BCAT1 as a potential therapy for TKI-resistant NSCLC.

## Discussion

Although EGFR TKIs have revolutionized the field of EGFR-mutant NSCLC therapy, acquired resistance inevitably occurs due to tumor heterogeneity and adaptability. The mechanisms of acquired resistance are complex and can involve EGFR alterations (mutation/amplification), activations of bypass or downstream pathways, and changes in the phenotype.^5,6^ However, the contribution of metabolic reprogramming in EGFR-TKI resistance is poorly understood. Here, based on our previously established resistant NSCLC models, we identified BCAT1 as a crucial biomarker and mediator of drug resistance to third-generation EGFR TKIs, including osimertinib and ASK120067. Knockdown or pharmacological inhibition of BCAT1 selectively inhibited the in vitro and in vivo growth of resistant tumors and partially restored their sensitivity to EGFR TKIs. Mechanistically, elevated production of α-KG by BCAT1 promoted the expression of glycolysis genes by facilitating KDM6A/6B-mediated H3K27 demethylation, thus leading to enhanced glycolysis, cell survival, and drug tolerance. Consistent with these preclinical findings, BCAT1 and several key downstream glycolysis genes are upregulated in primary lung cancer, with even higher expression in samples from patients who eventually relapsed. Furthermore, BCAT1 expression is significantly higher in samples from TKI-resistant NSCLC patients compared to those from TKI-sensitive patients and is associated with poor prognosis in NSCLC. Our findings highlight the important role of BCAT1 in mediating metabolic reprogramming and drug resistance, indicating its promising potential as a biomarker and therapeutic target in NSCLC.

BCAT1 is a cytosolic enzyme that initiates the catabolism of BCAAs. Enhanced expression of BCAT1 disrupts BCAA metabolism and contributes to liver disease, inflammatory disease, insulin resistance, Alzheimer’s disease, and tumor progression.^14,28,29^ BCAT1 catalyzes the reversible transamination of BCAAs to BCKAs, accompanied by the amination of α-KG to glutamate, and these metabolites are involved in multiple cellular processes. Therefore, how BCAT1 reprograms BCAA metabolism to support tumor progression is complex and could be cell context- and stress context-dependent. In IDH1^wt^ gliomas, BCAT1 is activated to facilitate glutamate production for promoting cell proliferation, and catabolism of BCAAs is required to sustain tumor progression.^30^ In NSCLC, BCAT1 transcription is associated with poor overall survival of patients, and overexpression of BCAT1 causes α-KG restriction and further increases SRY (sex determining region Y) -box transcription factor 2 (SOX2) expression to promote cell stemness and metastasis.^31^ Moreover, upregulated BCAT1 in lung cancer cells has been shown to enhance GSH production, contributing to short-term acquired resistance to the first-generation EGFR TKI gefitinib.^15^ Recently, a gain-of-function BCAT1 glutamic acid to alanine mutation at codon 61 (BCAT1^E61A^) in gastric cancer was identified in clinical cases. It confers higher enzymatic activity to boost BCAA catabolism and accelerate cell growth and motility and contributes to tumor development by BCKA-dependent elevation of RhoC activity.^32^ The above studies support the oncogenic roles of BCAT1 by facilitating BCAA breakdown and increasing downstream catabolic metabolites. Additionally, opposing metabolic fluxes promoted by BCAT1 have also been reported. In chronic myeloid leukemia (CML), BCAT1 is aberrantly activated and required for tumor propagation by promoting BCAA production.^13^ Also, BCAT1 is reported to cooperate with NRAS G12D to sustain intracellular BCAA pools and promote leukemic transformation through activated mTOR signaling.^33^ In our study, we demonstrated that BCAT1 facilitated BCAA anabolic metabolism, thereby enhancing α-KG-dependent glycolysis to orchestrate resistance to third-generation EGFR TKIs in NSCLC. This finding provided a new context-dependent mechanism of BCAA metabolism reprogramming-mediated tumor progression and further elucidated the role of BCAT1 in conferring EGFR-TKI resistance. However, the drivers of BCAT1 upregulation during acquired drug resistance were not discussed in this study and deserves further investigation.

α-KG is an essential metabolite involved in various metabolic and epigenetic regulatory processes and shows multifaceted contributions to tumor progression. Considering only its role in chromatin modifications, α-KG can cause distinct cellular effects through its predominant downstream genes under different circumstances. For example, the accumulation of α-KG induced by restoring p53 function upregulates chromatin modification of 5-hmC to promote tumor suppressor gene expression in cancer cells derived from *Kras* mutant pancreatic ductal adenocarcinoma (PDAC).^34^ In contrast, elevated α-KG is reported to be required to maintain an epigenetic state of low H3K27me3 by serving as a cofactor for the H3K27 demethylases KDM6A/6B in diffuse midline gliomas (DMGs) bearing driver mutations of histone 3 lysine 27 (H3K27M).^35^ In our TKI-resistant NSCLC models, we reveal that α-KG is a key metabolite upregulated by BCAT1, epigenetically activating glycolysis and sustaining cell survival and drug resistance. Although an activated glycolytic phenotype and non-mutational epigenetic reprogramming are emerging as important cancer hallmarks, their roles in third-generation EGFR-TKI resistance are not yet fully established.^36,37^ Our findings present the function of α-KG in linking BCAA metabolism to epigenetic regulation and glycolysis in TKI-resistant NSCLC, which provides an extensive understanding of the intricate crosstalk between multiple metabolic pathways and epigenetics in cancer development.

Increasing evidence implicates BCAT1 as a promising drug target for cancer therapy, yet the discovery of effective BCAT1 inhibitors remains limited. Among these inhibitors, gabapentin is the most commonly used BCAT1 inhibitor, which has demonstrated in vitro or in vivo antitumor efficacies in glioblastoma, hepatocellular carcinoma, and CML.^13,30,38,39^ Additional compounds, such as BCATc inhibitor 2, ERG240, tubercidin, lycorine HCl, and BAY-069 also shown promising BCAT1-targeting activities.^40–42^ While BCATc inhibitor 2 and ERG240 have demonstrated neuroprotective effects or alleviated autoimmune diseases in murine models, their antitumor efficacy remains unexplored. Tubercidin and lycorine HCl have exhibited prominent in vitro and in vivo anti-growth effects on small-cell lung cancer (SCLC). BAY-069, targeting both BCAT1 and BCAT2, has exhibited significant antiproliferative effects on glioblastoma cells and breast cancer cells. These research findings underscored the therapeutic implications of targeting BCAT1 not only for inflammatory disease and also for multiple tumors. However, none of these inhibitors has shown strong antitumor potency in NSCLC. In this study, we identified WQQ-345 as a novel BCAT1 inhibitor that selectively suppressed the in vitro growth of BCAT1-highly expressing third-generation EGFR TKI-resistant cells and exerted promising antitumor efficacy in resistant xenograft model. Additionally, ongoing efforts are focused on developing more selective BCAT1 inhibitors with enhanced efficacy and evaluating their performance in established tumor models.

In summary, this study elucidates the significance of BCAT1-mediated BCAA metabolic reprogramming in enhancing cell survival and TKI resistance in NSCLC, which provided extensive evidence for directly targeting BCAT1 as a therapeutic strategy for lung cancer.

## Materials and methods

### Cell culture and compound reagents

Human NSCLC cell lines NCI-H1975 and PC9 were obtained from the American Type Culture Collection (ATCC). ASK120067-resistant cells (67R) and osimertinib-resistant cells (AZDR) were established, maintained, and authenticated as described previously.^3^ EGFR Exon 19del/T790M expressing BaF3 (BaF3-EGFR^19D/T790M^) cell lines were established using retrovirus-mediated gene overexpression. All cell lines were cultured in RPMI 1640 medium supplemented with 10% FBS in a humidified cell culture incubator at 37 °C with 5% CO_2_.

ASK120067 was provided by Aosaikang Pharmaceutical (Nanjing, China). Osimertinib (#S7297) was obtained from Selleck (Houston, USA). Gabapentin (#T0702) was purchased from TargetMol (Boston, USA). GSK-J4 hydrochloride (GSK-J4 HCI, #CSN19181) and Bobcat339 (#CSN24474) were purchased from CSNpharm (Chicago, USA). Dimethyl 2-oxoglutarate (DM-αKG, #349631) was obtained from Sigma-Aldrich (St. Louis, USA).

### Stable isotope labeling with amino acids in cell culture (SILAC) assay

Detailed information on mass spectrometry proteomic analysis is shown in the Supplementary materials and methods.

### Bioinformatic analysis

The GSE31210 dataset was obtained from the Gene Expression Omnibus (GEO) database (https://www.ncbi.nlm.nih.gov/geo/), which contained 226 patients with primary stage I–II lung adenocarcinomas and 20 normal lung tissues.^43^ The expression profile data were analyzed using “GEOquery” package in R environment.^44^ The patients were grouped based on median expression of BCAT1 or BCAT2. Differential expression genes (DEGs) analysis by “Limma” package was subsequently conducted between different groups.^45^ Prognosis analysis was conducted using the R package “Survival”, and Gene Set Enrichment Analysis (GSEA) was performed using “clusterprofiler” R package.^46^ The GSE231938 dataset acquired from the GEO database provided RNA-Seq data in 2 TKI-sensitive NSCLC patients and 2 TKI-resistant patients.^47^

### Western blotting

Cells were lysed using SDS lysis buffer, and the protein concentrations were determined using Pierce BCA protein assay kit (Thermo Fisher Scientific, 23225) for normalization. Tumor samples were lysed in RIPA buffer supplemented with protease inhibitor cocktail and phosphatase inhibitor (Roche). After quantification, proteins were separated by SDS-PAGE gel and transferred to nitrocellulose membranes. Membranes were blocked with 5% non-fat milk-TBST for 1 h at room temperature and then incubated overnight at 4 °C with diluted primary antibodies against BCAT1 (Cell Signaling Technology, #12822S), BCAT2 (Cell Signaling Technology, #9432), β-Actin (Proteintech, #66009-1-Ig), H3K79me2 (PTM BIO, #PTM-5159), H3K36me3 (Cell Signaling Technology, #4909S), H3K27me3 (Cell Signaling Technology, #9733S), H3K27me2 (Cell Signaling Technology, #9728S), H3K27me1 (PTM BIO, #PTM-649), H3K9me3 (Abclonal, #A2360), H3K9me2 (PTM BIO, #PTM-615), H3K4me3 (Cell Signaling Technology, #9751S), H3K4me2 (Cell Signaling Technology, #9725S), H3K4me1 (Cell Signaling Technology, #5326S), H3 (Cell Signaling Technology, #4499S), α-Tubulin (Proteintech, #66031-1-Ig), GAPDH (Proteintech, #60004-1-Ig), PFKP (Cell Signaling Technology, #8164S), LDHA (Cell Signaling Technology, #3582S), PKM1/2 (Cell Signaling Technology, #3190S). The membranes were washed with TBS-T and then incubated with secondary antibodies. After another wash with TBS-T, immunoreactive bands were visualized using chemiluminescence (Thermo Fisher Scientific). The relative levels of protein expression were quantified using ImageJ software and were normalized with either β-Actin, Histone H3 or α-Tubulin.

### RT-qPCR

Total RNA was extracted from cells by EZ-press RNA Purification Kit (EZBioscience, #B0004D). Reverse transcriptase reaction was performed with HiScript II Q RT SuperMix for qPCR (Vazyme, #R223-01). Quantitative real-time PCR was performed using ChamQ Universal SYBR qPCR master mix (Vazyme, #Q711-02). Target gene expression levels were normalized against β-Actin mRNA level. All primer sequences used for different genes are listed in Supporting Information: Table S1.

### Immunohistochemistry

Fixed sections of clinical lung cancer tumors, adjacent noncancerous tissues and TKI-resistant lung cancer tumors were obtained from Shanghai Chest Hospital, Shanghai Jiao Tong University, and Shanghai Institute of Biochemistry and Cell Biology, Chinese Academy of Sciences. Study with these clinical samples is approved by the institutional review committees of Ethics Committee of Shanghai Chest Hospital (Approval number: IS23049), and Shanghai pulmonary hospital, Tongji University (Approval number: K23-171), respectively. Immunohistochemistry of paraffin sections was carried out by Shanghai ZuoChengBio using a two-step protocol according to the manufacturer’s instructions. Briefly, paraffin sections were first deparaffinized with xylene and then hydrated with ethanol. Antigen retrieval was performed by immersing the specimens in 0.01 mol/L citrate buffer at pH 6.0 and exposing it to microwave heating for 10 min at 450 W. Sections were then incubated overnight at 4–8 °C with primary antibody against BCAT1 (Proteintech, #13640-1-AP). After washing off the primary antibody, the sections were treated with the Envision detection system kit (EnVision1/HRP/Mo, Dako, Glostrup, Denmark). Reaction products were visualized by incubating with DAB. Finally, the sections were counterstained with hematoxylin (Sigma-Aldrich, Germany) to visualize the nuclei.

### Lentiviral—shRNA gene knockdown

BCAT1 targeted shRNA vectors (pLKD-CMV-G&PR-U6-shRNA) were purchased from Obio technology (Shanghai, China). Targeted sequences were as follows: shCtrl: 5′-TTCTCCGAACGTGTCACGT-3′; shBCAT1-1: 5′-GGGAGAAACCTCATATCAA-3′; shBCAT1-2: 5′-GGAGAAGAAGAACTGGCAA-3′. shBCAT1 was the same as shBCAT-1. Lentivirus was produced by transfecting 293 T cells with Lipofectamine 2000 using shRNA vectors and lentiviral packaging plasmids (pCMV-Gag-Pol and pCMV-VSV-G). 293T culture supernatants containing viral particles were collected at 48 h after transfection and filtrated through a 0.45 μm filtration membrane. Cells were infected with shRNA-lentivirus with 8 μg/mL polybrene for 24 h, and positive clones were obtained by puromycin selection.

### Colony formation assays

Cells were seeded into a 6-well or 12-well plate at a density of 800–1000 cells per well and cultured overnight. After being exposed to indicated agents for 7 to 10 days, cells were fixed with fixation fluid (10% acetic acid + 10% methanol + 80% ddH_2_O) and stained with crystal violet. The colonies were finally imaged and counted.

### Cell proliferation assays

Cell proliferation was evaluated using the sulforhodamine B (SRB) colorimetric assay. Cells were seeded into 96-well plates and cultured overnight. Then cells were treated with indicated concentration of test compounds for 72–108 h. Subsequently, the SRB assay was conducted according to standard protocols Specifically, 100 μL of 10% pre-cooled trichloroacetic acid (TCA) was added to each well after removing the culture medium. The plate was then incubated for 1 h at 4 °C to fix the cells. After incubation, the TCA solution was removed, and the wells were washed five times with distilled water. After the plates dried, 100 μL of 4 mg/mL SRB solution (Sigma, St Louis, MO, USA) prepared with 1% acetic acid was added to each well and incubated for 15 min at room temperature. Then the SRB solution was removed, and each well was washed five times with a 1% acetic acid solution before drying. To solubilize the stain, 150 µL of 10 mmol/L Tris-HCl was added to each well, and the plate was shaken occasionally at room temperature. The absorbance at 560 nm of each well was then measured using a multi-well VersaMax spectrophotometer (Molecular Devices, Sunnyvale, CA, USA). The proliferation inhibition rates were calculated as follows: Inhibition rate (%) = [1 − (A560 _treated_/A560 _control_)] × 100%. The IC_50_ values were calculated using the Logit method based on dose–response curve. Additionally, the effects of drug combination were visualized, and synergy scores were analyzed by SynergyFinder using Bliss model. The interaction between two drugs is considered as synergistic, additive or antagonistic when synergy score is more than 10, from −10 to 10, or less than −10, respectively.

### In vivo xenograft model and drug administration

67R cells (1 × 10^7^ cells per mouse), 67R-shCtrl cells (5 × 10^6^ cells per mouse), or 67R-shBCAT1 cells (1 × 10^7^ cells per mouse) were subcutaneously implanted into the right flanks of 4-week-old BALB/c nude mice. Tumors with stable morphology were then cut into fragments and transplanted into the right flanks of nude mice. When tumors reached nearly 50–70 mm^3^, mice were randomly divided into control and treatment groups. Tumor volume (TV) was measured and calculated using the formula TV = (L × W^2^)/2. L and W represented the longest and shortest diameter (mm) of the tumor, respectively. The tumor growth inhibition rate (TGI) was calculated as TGI (%) = [1 – (T_Vt_ – T_V0_)/(C_Vt_ − C_V0_)] × 100. T_vt_ and C_Vt_ were the average tumor volumes on the day of investigation in the treatment group and control group, respectively. T_V0_ and C_V0_ represented the tumor volume of the treatment group and control group at the beginning of the study, respectively. All procedures on mice were performed according to the guidelines approved by the Institutional Animal Care and Use Committee (Approval number: 2022-02-DJ-66) following the guidance of the Association for Assessment and Accreditation of Laboratory Animal Care at Shanghai Institute of Materia Medica.

### ^13^C-Stable isotope labeling

Cells were seeded in 10 cm plates and cultured overnight in RPMI 1640 complete medium. After being washed three times with PBS, the cells were incubated with custom RPMI 1640 medium lacking BCAAs with supplementation of [^13^C]- leucine_M + 6 (50 mg/L), isoleucine (50 mg/L), valine (20 mg/L), [^13^C] KIC M + 2 (20 μM) and 10% FBS for 2 h. After that, cells were digested using trypsin-EDTA and washed 3 times with PBS. Cell pellet samples (10^7^ cells/sample) were collected and stored at −80 °C. Gibco® custom RPMI 1640 medium lacking BCAAs were purchased from Thermo Fisher Scientific. [^13^C]-Leucine_M + 6 (#CLM-2262-H-0.1) and [^13^C]-KIC_M + 2 (#CLM-4826-0.1) were purchased from Cambridge Isotope Laboratories. Isoleucine (#I7403) and valine (#V0513) were obtained from Sigma-Aldrich (St. Louis, USA).

Labeled metabolites were quantitated by Metabo-Profile Biotechnology (Shanghai, China). Briefly, cell samples were added with 400 μL of 80% methanol solution and sonicated (JY92-IIN, NingBo Scientz Biotechnology Co., Ltd.). After centrifugation and concentration, the supernatant was added with 100 μL of 80% methanol solution. Metabolite analysis was conducted using an ultrahigh-pressure liquid chromatography-triple quadrupole mass spectrometer, ACQUITY UPLC-Xevo TQ-S (Waters Corp., Milford, MA, USA). The raw data files generated by UPLC-MS/MS were processed using MassLynx software (v 4.1, Waters Corp., Milford, MA, USA) for peak extraction, integration, identification, and quantification of each metabolite. R language (v4.1.1) was employed for subsequent statistical analysis.

### Metabolite detection assays

Intracellular BCAAs and alpha-ketoglutarate (α-KG) were measured using Branched Chain Amino Acid (Leu/Ile/Val) Colorimetric Assay Kit (Biovision, K564-100) and Alpha Ketoglutarate (alpha KG) Assay Kit (Abcam, ab83431) respectively, following the manufacturer’s instructions. The absorbance was measured using a microplate reader to determine metabolite concentrations. Normalization of metabolite was conducted based on cell number.

### RNA interference

The specific small interfering RNA (siRNA) targeting BCAT1 (#siB1198165207, #stB0005685C) and negative control siRNA (#siN0000001-1-5) were purchased from RiboBio (Guangzhou, China). Cells were transfected using siRNA with Lipofectamine RNAiMAX (Thermo Fisher Scientific) according to the manufacturer’s instructions. Targeted sequences for BCAT1 were as follows, siBCAT1-1: 5′- GTACAAAGGCGAGACAATA-3′, siBCAT1-2/ siBCAT1: 5′- CAAGCCGCATCTTGAGCAA-3′.

### RNA sequencing (RNA-seq)

Total RNA was extracted using TRIzol® Reagent (Invitrogen, #15596018) following the instructions provided by the manufacturer. Samples were sent to Shanghai Majorbio Bio-pharm Biotechnology Co., Ltd. (Shanghai, China) to perform RNA purification, reverse transcription, library construction and sequencing. The transcriptome library for RNA-seq was generated by using 1 μg of total RNA with the Illumina® Stranded mRNA Prep, Ligation kit from Illumina (San Diego, CA). After quality control of raw reads, the clean reads were mapped using HISAT2 software and further assembled by StringTiein. Cellular pathway analysis was conducted using GSEA.

### Extracellular acidification rate (ECAR) measuring

Seahorse XF Glycolytic Rate Assay Kit (Agilent Technologies, #103344-100) was used to measure real-time extracellular acidification rate (ECAR) according to the manufacturer’s protocol. Cells were initially seeded in the Agilent Seahorse XFp Cell Culture Miniplate and cultured overnight. Subsequently, the cells were washed and cultured in Seahorse XF RPMI medium (Agilent Technologies, #103576-100) at pH 7.4, supplemented with 1 mM pyruvate, 10 mM glucose, and 2 mM glutamine. The cells were then incubated at 37 °C in a non-CO_2_ incubator for 45–60 min and followed by exposure to 0.5 μM Rotenone and Antimycin A (Rot/AA), 50 mM 2-Deoxy-d-glucose (2-DG) sequentially. Finally, the assay template was loaded onto the Seahorse XFp Analyzer, and real-time ECAR was recorded.

### Chromatin immunoprecipitation (ChIP) assay

ChIP assays were performed using SimpleChIP® Plus Enzymatic Chromatin IP Kit (Cell Signaling Technology, #9005S) according to the manufacturer’s instructions. Samples were sonicated and immunoprecipitated using either IgG or anti-H3K27me3 antibody (Cell Signaling Technology, #9733 S). The levels of immunoprecipitated DNA fragments were evaluated by qPCR. Primers used are listed in Supporting Information: Table S2.

### DNA dot blot

Total DNA samples were extracted using the AFTSpin Blood/Tissue/Cell Fast DNA Extraction Kit (Abclonal, #RK30110) and denatured at 95 °C for 10 min. Samples were rapidly placed on ice for 10 min and then loaded to positively charged nylon membranes. After being crossed with UV, the membranes were blocked with 5% non-fat milk for 1 h and incubated with 5 mC (Abclonal, #A20599) and 5hmC antibodies (Cell Signaling Technology, #51660S). Besides, the membranes were stained with 0.2% methylene blue to verify equal DNA input.

### Chemistry

The synthetic procedures and characterization of the compound against BCAT1 were provided in the Supporting Information.

### BCAT1 enzyme activity assay

BCAT1 inhibitor was preincubated with 40 ng recombinant human BCAT1 Protein (Sino Biological, #ME16FE2265) in assay buffer (50 mM Tris, 0.05% Tween-20, pH 8.0) at 37 °C for 30 min and finally consisted 10 μL reaction mix. The substrate mixture, comprising 1U GIDH, 2 mM β-NAD, 0.04 mM Resazurin, 0.5 mM alpha-Ketoglutaric Acid, 0.05 mM Leucine, and 11 ng rhNQO-1 (Sigma, #N6522) were subsequently added to the reaction mix. The rate of increase in fluorescence was immediately monitored and used for the calculation of IC_50_ values.

### Molecular Docking study

The docking studies were performed using AutoDock Vina (version 1.1.2) and AutoDockTools (version 1.5.7).^48,49^ The crystal structure of BCAT1 was obtained from RCSB Protein Data Bank (PDB code: 2COJ). The docking site was defined using the program AutoGrid with a grid box size of 15 Å × 15 Å × 15 Å (x, y, z), spacing of 0.375 Å, and grid center of −9.7 Å (x), 8.9 Å (y), and −7.0 Å (z). Figures were generated with PyMOL (version 1.3).

### Cellular thermal shift assay (CETSA)

Cells were treated with either compound or PBS for 4 h and collected. After being washed twice with PBS, the cell suspension was aliquoted and heated at 58, 62, 64, and 66 °C for 3 min. Subsequently, cells were lysed with three repeated freeze-thaw cycles using liquid nitrogen. The supernatants were obtained by centrifugation at 12,000 *g* for 20 min and diluted with 5 × SDS loading buffer. The resultant proteins were boiled at 100 °C for 20 min and analyzed by Western blot assay.

### Statistical analysis

Statistical analyses were performed by GraphPad Prism 8.0. The results were presented as mean ± SD from at least three independent experiments. Mean ± SEM was utilized to present data on tumor volume. Comparisons between the two groups were analyzed using Student’s *t* test. One-way or two-way analysis of variance (ANOVA) was employed for comparisons involving multiple groups and/or conditions. *p* < 0.05 was considered as statistically significant.

### Supplementary information

Supplementary Materials

Dataset 1

## Data Availability

The mass spectrometry proteomics data have been deposited to the integrated proteome resources (iProX) (https://www.iprox.cn/) via the PRIDE partner repository with the identifier IPX0006742000 or PXD043776.^50^ The RNA-sequencing data has been deposited in Gene Expression Omnibus under accession code GSE247584. All data in the current study are available upon reasonable request from the corresponding authors.
