# Supplementary material for: Branched-chain amino acid transaminase 1 confers EGFR-TKI resistance through epigenetic glycolytic activation
Source: Signal Transduct Target Ther. 2024 Aug 15;9:216. doi: 10.1038/s41392-024-01928-8 (PMC11324870; doi:10.1038/s41392-024-01928-8)
Supplement: Supplementary file 1 — Supplementary Materials [file 41392_2024_1928_MOESM1_ESM.docx]

Supplementary Materials for

**Branched-chain Amino Acid Transaminase 1 Confers EGFR-TKI (Tyrosine Kinase Inhibitor) Resistance through Epigenetic Glycolytic Activation**

Tao Zhang^1†^, Zilu Pan^1,3†^, Jing Gao^1†^, Qingqing Wu^2†^, Gang Bai^1^, Yan Li^1^, Linjiang Tong^1^, Fang Feng^1^, Mengzhen Lai^1^, Yingqiang Liu^1^, Peiran Song^2^, Yi Ning^1^, Haotian Tang^1^, Wen Luo^2,5^, Yi Chen^1,3^, Yan Fang^1,3^, Hui Zhang^6^, Qiupei Liu^1,7^, Yudi Zhang^1,3,8^, Hua Wang^3,8^, Zhiwei Chen^1,3^, Yi Chen^1^, Meiyu Geng^1,3,4^, Hongbin Ji^3,8^, Guilong Zhao^1,2,5*^, Hu Zhou^1,3*^, Jian Ding^1,3,4*^ and Hua Xie^1,2,3*^

*Correspondence: Guilong Zhao ([zhaoguilong@simm.ac.cn](mailto:zhaoguilong@simm.ac.cn)) or Hu Zhou ([zhouhu@simm.ac.cn](mailto:zhouhu@simm.ac.cn)) or Jian Ding ([jding@simm.ac.cn](mailto:jding@simm.ac.cn)) or Hua Xie ([hxie@simm.ac.cn](mailto:jding@simm.ac.cn))

**This PDF file includes:**

Materials and Methods

Figures. S1 to S28

Tables S1 to S2

**Materials and Methods**

Stable isotope labeling with amino acids in cell culture (SILAC) Assay

*Materials*

Ammonium bicarbonate, dithiothreitol (DTT), iodoacetamide (IAA), sodium carbonate, L-arginine (Arg-0), L-lysine (Lys-0), L-^13^C_6_-arginine (Arg-6), L-^2^H_4_-lysine (Lys-4), L-^13^C_6_^15^N_4_-arginine (Arg-10) and L-^13^C_6_^15^N_2_-lysine (Lys-8) were purchased from Sigma-Aldrich (Saint Louis, MO). SILAC RPMI-1640 medium and dialyzed FBS were purchased from Thermo Fisher Scientific (Waltham, MA). Acetonitrile and water for nano-LC-MS/MS were purchased from J.T. Baker (Phillipsburg, NJ). Trypsin was purchased from Promega (Madison, WI). 10k Microcon filtration device was purchased from Millipore (Billerica, MA).

*Protein trypsin digestion using FASP Method*

NCI-H1975, AZDR and 67R cell lines were labeled with Lys-0Arg-0 (“light”), Lys-4Arg-6 (“medium”) and Lys-8Arg-10 (“heavy”), respectively, by culturing them in SILAC RPMI-1640 medium supplemented with 10% (vol/vol) dialyzed serum. After eight passages, the full incorporation of SILAC amino acids was confirmed. Cells were washed with PBS three times and lysed with SDT buffer (2%SDS, 100 mM Tris, 100 mM DTT, pH = 7.5). Lysates were incubated at 95°C for 5 min. Protein concentrations were determined by tryptophan fluorescence emission at 350 nm using an excitation wavelength of 295 nm as previously described^1^.

Equal protein amounts of the three cell lines were combined and diluted in 8 M urea in 0.1 M Tris-HCl (pH 8.0). 100μg of sample was loaded on a 10k Microcon filtration device and digested by the FASP procedure as previously described^2^.

*High pH reversed phase Liquid Chromatography peptide fractionation*

Peptide mixture from biological replicates was fractionated using a Waters XBridge BEH130 C18 3.5 μm 2.1 × 150 mm column on an Agilent 1290 HPLC (*Santa Clara*, CA) operating at 0.2 mL/min as previously described^3^. The total number of fractions concatenated was set to 14 throughout all experiments.

*Liquid Chromatography tandem mass spectrometry (LC-MS/MS)*

The reversed-phase high-performance liquid chromatography (RP-HPLC) separation was achieved on the Easy nano-LC1000 system (Thermo Fisher Scientific) using a self-packed column (75μm × 150mm; 3 μm ReproSil-Pur C18 beads, 120 Å, Dr.Maisch GmbH, Ammerbuch, Germany) at a flow rate of 300 nL/min. The mobile phase A of RP-HPLC was 0.1% formic acid in water, and B was 0.1% formic acid in acetonitrile. The peptides were eluted using a gradient (2–90% mobile phase B) over a 90 min period into a nano-ESI Q Exactive mass spectrometer (Thermo Fisher Scientific). The mass spectrometer was operated in data-dependent mode with each full MS scan (m/z 350 - 1600) followed by MS/MS for the 15 most intense ions with the parameters: +2-+4 precursor ion charge, 2 Da precursor ion isolation window and 25 normalized collision energy of HCD. Dynamic Exclusion™ was set for 40 s. The full mass and the subsequent MS/MS analyses were scanned in the Orbitrap analyzer with R = 70,000 and R = 17,500, respectively.

*Data analysis*

The MS data were analyzed using the software MaxQuant (http://maxquant.org/, version 1.5.3.30)^4^. Proteins were identified by searching MS and MS/MS data of peptides against a human proteome database from Uniprot (2016.02.15 downloaded). Trypsin/P was selected as the digestive enzyme with two potential missed cleavages. The search included variable modifications of methionine oxidation and N-terminal acetylation, and fixed modification of cysteine carbamidomethylation. Peptides of minimum 6 amino acids were allowed for the analysis. For peptide and protein identification, false discovery rate was set to 0.01. Proteins were considered as significantly differentially expressed between experimental groups if they displayed at least a 1.5-fold change in their corresponding mean of SILAC ratios. Hierarchical clustering of proteins was performed using Euclidean distances and Ward clustering method by Package of ‘pheatmap’ in language R. Gene Ontology annotation and pathway analysis was performed by Package of ‘Gostat’ in language R^5^.

Chemistry

Compound **WQQ-345** was synthesized by a route shown in Scheme 1. Thus, starting tricyclic alcohol (±)-**1**^6^ was oxidized to corresponding ketone (±)-**2** by Swern oxidation in dried CH_2_Cl_2_ at -78°C to room temperature, which was subsequently subjected to Wittig condensation reaction with *tert*-butyl diethylphosphonoacetate in the presence of *t*-BuOK in dried THF at 0°C to room temperature to afford α,β-unsaturated carboxylic ester (±)-**3** as a mixture of *E*/*Z* isomers. Treatment of (±)-**3** with 1,8-diazabicyclo[5.4.0]undec-7-ene (DBU) in CH_3_NO_2_ at 80°C-85°C afforded (±)-**4** via Michael addition. Ester (±)-**4** was hydrolyzed with NaOH in aqueous ethanol at reflux to afford corresponding acid (±)-**5**, which was finally hydrogenated on Pd(OH)_2_ as catalyst in methanol at room temperature to produce desired **WQQ-345** by reduction of nitro group and concomitant opening of the cyclopropane in a stereoselective manner. It should be noted that **WQQ-345** was in a form of racemic mixture.

*General*

Melting points were measured with an SGW X-4A microscopic melting point apparatus (Shanghai INESA Physico-Optical Instrument Co., Ltd., Shanghai, China) and are uncorrected. NMR spectra were recorded on a Bruker Avance III 500, Bruker Avance III 600, or Bruker Avance III HD 600 NMR spectrometer using CDCl_3_, CD_3_OD or D_2_O as solvent and TMS (for ^1^H NMR) or known chemical shifts of carbon signals of deuterated solvents (for ^13^C NMR) as internal standard. High-resolution mass spectra (HR-MS) were recorded with a Thermo Q Exactive Plus mass spectrometer (Thermo Fisher Scientific, Bremen, Germany) using electrospray ionization (ESI) technique. All the dried solvents were prepared by known standard methods.

*Detailed description of the synthesis of WQQ-345*

(1) Synthesis of (*1R**,*2R**,*7S**)-tricyclo[3.2.0.0^2,7^]heptan-4-one ((±)-**2**)

To a stirred solution of dried dimethylsulfoxide (53.28 g, 0.68 mol) in dried CH_2_Cl_2_ (300 mL) cooled at -78℃ was added dropwise a solution of (COCl)_2_ (43.15 g，0.34 mol) in dried CH_2_Cl_2_ (200 mL). The addition is vigorously exothermic and therefore the addition rate should be adjusted so that the internal temperature was kept below -70℃. After addition, the stirring was continued for another 0.5 h at this temperature followed by dropwise addition of a solution of (±)-**1** (27.01 g, 0.25 mol) prepared following the reported procedure^6^ in dried CH_2_Cl_2_ (300 mL). After addition, the stirring was continued for another 1 h at this temperature followed by dropwise addition of Et_3_N (114.15 g, 1.13 mol). After addition, the resulting mixture was stirred at this temperature for 1 h and then gradually warmed to room temperature followed by stirring at room temperature for another 10 h. The reaction mixture was washed successively with 1 M HCl (800 mL), water (800 mL) and saturated aqueous NaHCO_3_ (800 mL), dried (MgSO_4_) and evaporated on a rotary evaporator to afford crude (±)-**2** as a colorless oil, which was not very stable and was used directly in the next step without further purification and extensive characterization. ^1^H NMR (600 MHz, CDCl_3_) *δ* 2.75-2.79 (m, 1H), 2.64-2.67 (m, 2H), 2.55-2.59 (m, 1H), 2.49-2.52 (m, 1H), 1.85-1.87 (m, 1H), 1.71-1.73 (m, 1H), 1.56 (dd, 1H, *J* = 3.0 and 10.8 Hz). ^13^C NMR (151 MHz, CDCl_3_) *δ* 218.60, 47.07, 34.31, 26.65, 23.64, 17.74, 17.23. ESI-HRMS: (m/z) calcd for C_7_H_9_O ([M+H]^+^) 109.0648, found 109.0651.

(2) Synthesis of *tert*-butyl (*E*/*Z*)-2-((*1R**,*2R**,*7S**)-tricyclo[3.2.0.0^2,7^]heptan-4-ylidene)acetate ((±)-**3**)

To a stirred suspension of *t*-BuOK (41.12 g, 0.37 mol) in dried THF (200 mL) cooled on an ice-water bath was added dropwise *tert*-butyl diethylphosphonoacetate (91.22 g, 0.36 mol). After addition, the resulting mixture was stirred at this temperature for another 0.5 h, followed by dropwise addition of a solution of (±)-**2** prepared above in dried THF (60 mL). After addition, the reaction mixture was stirred at this temperature until the reaction completed as indicated by TLC analysis (typically within 1 h) and poured into stirred ice-water (300 mL). The resulting mixture was extracted with EtOAc (100 mL × 2), and the combined extracts were washed with brine, dried (MgSO_4_) and evaporated on a rotary evaporator to afford a residue, which was purified by column chromatography to afford (±)-**3** as a mixture of *E*/*Z* isomers. Colorless oil, 30.25 g (overall yield of 60% from (±)-**1** to (±)-**3**). This product was used directly in the next step without further purification and characterization.

(3) Synthesis of *tert*-butyl (*E*/*Z*)-2-((*1R**,*2R**,*4R**,*7S**)-4-(nitromethyl)tricyclo[3.2.0.0^2,7^]heptan-4-yl)acetate ((±)-**4**)

A mixture of (±)-**4** (30.25 g, 0.15 mol) and DBU in CH_3_NO_2_ (320 mL) was stirred at 80°C-85°C for 15 h when TLC analysis indicated that the reaction was essentially completed. On cooling to room temperature, the reaction mixture poured into stirred ice-water (500 mL), and the resulting mixture was extracted with CH_2_Cl_2_ (200 mL × 2). The combined extracts were washed with brine, dried (MgSO_4_) and evaporated on a rotary evaporator to afford an oily residue, which was purified by column chromatography to produce (±)-**4**. Colorless oil, 36.08 g (92%). ^1^H NMR (600 MHz, CDCl_3_) *δ* 4.63 (d, 1H, *J* = 10.8 Hz), 4.53 (d, 1H, *J* = 10.8 Hz), 2.44 (d, 1H, *J* = 16.2 Hz), 2.39 (d, 1H, *J* = 16.8 Hz), 2.36-2.39 (m, 1H), 2.31-2.33 (m, 1H), 2.20-2.24 (m, 1H), 1.98 (ddd, 1H, *J* = 1.8 Hz, 5.4 Hz and 13.8 Hz), 1.83 (d, 1H, *J* = 13.8 Hz), 1.53-1.56 (m, 1H), 1.45 (s, 9H), 1.42-1.47 (m, 1H), 1.32-1.34 (m, 1H). ^13^C NMR (126 MHz, CDCl_3_) *δ* 171.13, 81.05, 78.15, 52.00, 43.82, 35.83, 32.55, 28.24, 24.69, 22.12, 20.88, 16.38. ESI-HRMS: (*m/z*) calcd for C_14_H_22_NO_4_ ([M+H]^+^) 268.1543, found 268.1540.

(4) Synthesis of 2-((*1R**,*2R**,*4R**,*7S**)-4-(nitromethyl)tricyclo[3.2.0.0^2,7^]heptan-4-yl)acetic acid ((±)-**5**)

A mixture of (±)-**4** (36.01 g, 0.13 mol), NaOH (25.22 g, 0.63 mol), water (90 mL) and EtOH (400 mL) was refluxed until completion of reaction as indicated by TLC (typically within 5 h). On cooling to room temperature, the reaction mixture was poured into a stirred mixture of cooled 1 M HCl (1000 mL) and CH_2_Cl_2_ (300 mL), and the organic phase was separated. The aqueous phase was extracted with CH_2_Cl_2_ (200 mL), and the combined extracts were washed with brine, dried (MgSO_4_) and evaporated on a rotary evaporator to afford a residue, which was purified by trituration with *n*-hexane (200 mL) at room temperature to produce (±)-**5** after drying *in vacuo* at room temperature. White solid, 22.52g (79%). M.p. 103.5°C-105.5°C. ^1^H NMR (600 MHz, CD_3_OD) *δ* 4.66 (d, 1H, *J* = 11.4 Hz), 4.4.59 (d, 1H, *J* = 11.4 Hz), 2.54 (d, 1H, *J* = 16.8 Hz), 2.45 (d, 1H, *J* = 16.8 Hz), 2.39-2.42 (m, 1H), 2.30-2.32 (m, 1H), 2.20-2.24 (m, 1H), 1.98-2.02 (m, 1H), 1.80 (d, 1H, *J* = 13.8 Hz), 1.53-1.56 (m, 1H), 1.44-1.47 (m, 1H), 1.39-1.42 (m, 1H). ^13^C NMR (126 MHz, CD_3_OD) *δ* 174.99, 79.14, 52.68, 45.03, 35.18, 33.25, 25.56, 22.79, 21.82, 17.09. ESI-HRMS: (*m/z*) calcd for C_10_H_14_NO_4_ ([M+H]^+^) 212.0917, found 212.0917.

(5) Synthesis of 2-((*1R**,*2S**,*4S**)-2-(aminomethyl)bicyclo[2.2.1]heptan-2-yl)acetic acid (**WQQ-345**)

A mixture of (±)-**5** (5.05 g，24 mmol) and 10% Pd(OH)_2_/C (2.50 g) in MeOH (250 mL) was subjected to the standard hydrogenolysis procedure at atmospheric pressure (balloon) and room temperature until the reaction completed as indicated by TLC analysis (typically within 24 h). The reaction mixture was filtered off and the filtrate was evaporated on a rotary evaporator to afford a residue, which was triturated at room temperature in a mixed solvent consisting of MeOH (5 mL) and EtOAc (15 mL) to afford the target compound **WQQ-345** after drying *in vacuo* at room temperature. White solid, 3.15 g (72%). M.p. 173.6°C-176.0°C. ^1^H NMR (600 MHz, CD_3_OD) *δ* 2.94 (dd, 1H, *J* = 1.2 Hz and 13.2 Hz), 2.77 (d, 1H, *J* = 13.2 Hz), 2.62 (d, 1H, *J* = 16.2 Hz), 2.52 (dd, 1H, *J* = 1.2 Hz and 16.2 Hz), 2.36-2.37 (m, 1H), 2.25-2.26 (m, 1H), 1.75-1.79 (m, 1H), 1.57-1.66 (m, 2H), 1.44-1.50 (m, 1H), 1.33-1.37 (m, 1H), 1.30-1.32 (m, 1H), 1.22-1.26 (m, 1H), 1.06 (dd, 1H, *J* = 3.0 Hz and 12.6 Hz). ^13^C NMR (126 MHz, D_2_O) *δ* 181.55, 47.60, 46.37, 43.64, 41.50 (2C), 36.97, 36.70, 27.72, 24.21. ESI-HRMS: (*m/z*) calcd for C_10_H_18_NO_2_ ([M+H]^+^) 184.1332, found 184.1330. Purity: 97.09% (HPLC).

**Reference**

1 Geiger, T., Cox, J., Ostasiewicz, P., Wisniewski, J. R. & Mann, M. Super-SILAC mix for quantitative proteomics of human tumor tissue. *Nat. methods.* **7**, 383-385, (2010).

2 Wiśniewski, J. R., Nagaraj, N., Zougman, A., Gnad, F. & Mann, M. Brain Phosphoproteome Obtained by a FASP-Based Method Reveals Plasma Membrane Protein Topology. *J. Proteome Res.* **9**, 3280-3289, (2010).

3 Song, C. *et al.* Reversed-phase-reversed-phase liquid chromatography approach with high orthogonality for multidimensional separation of phosphopeptides. *Anal. Chem.* **82**, 53-56, (2010).

4 Cox, J. *et al.* A practical guide to the MaxQuant computational platform for SILAC-based quantitative proteomics. *Nat. Protoc.* **4**, 698-705, (2009).

5 Falcon, S. & Gentleman, R. Using GOstats to test gene lists for GO term association. *Bioinformatics (Oxford, England).* **23**, 257-258, (2007).

6 Last, L. A., Fretz, E. R. & Coates, R. M. Synthesis of polycyclic homocyclopropylcarbinols by reductive cyclization of bromocyclopropyl epoxides. *J. Org. Chem.* **47**, 3211-3219, (1982).


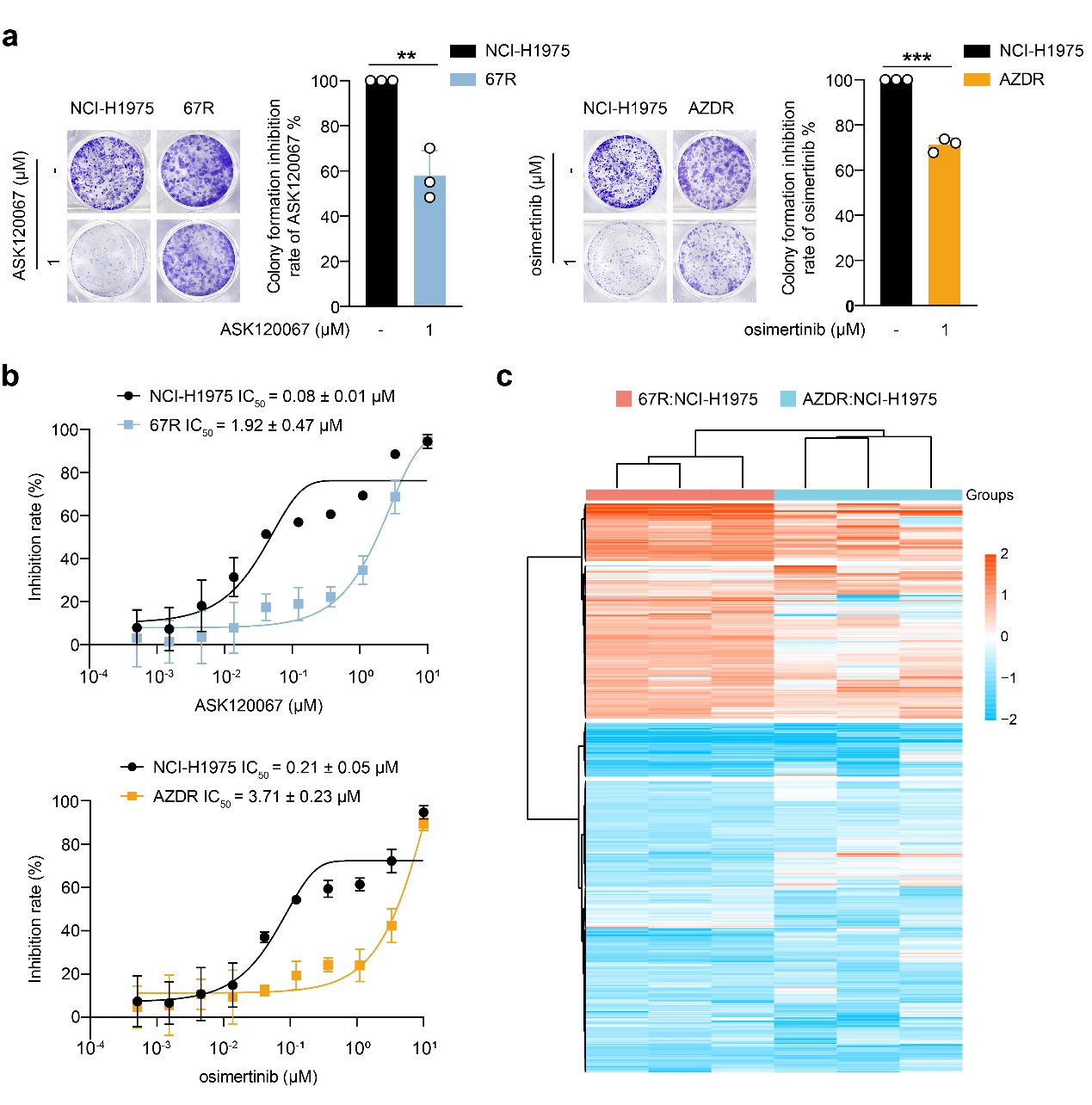


**Figure. S1.**

**Third-generation EGFR TKI-resistant cells exhibited distinct proteome patterns compared to parental NCI-H1975 cells.** **a** Colony formation assay of 67R, AZDR, and NCI-H1975 cells treated with ASK120067 or osimertinib. Representative images (left) and quantification results (right) are shown (n=3). **b** Anti-proliferation activities of ASK120067 or osimertinib in 67R, AZDR, and NCI-H1975 cells were evaluated using Sulforhodamine B assay. **c** Hierarchical cluster analysis of differential proteins (fold change ≥1.5) determined by SILAC-based proteomics in 67R or AZDR drug-resistant cell lines versus the parental NCI-H1975 cell lines is shown as a heatmap. The ratios of proteins were Log2 transformed and hierarchically clustered using the “ward” method with Euclidean distance. ***p <* 0.01, ****p <* 0.001. Data are expressed as the mean ± SD.

**
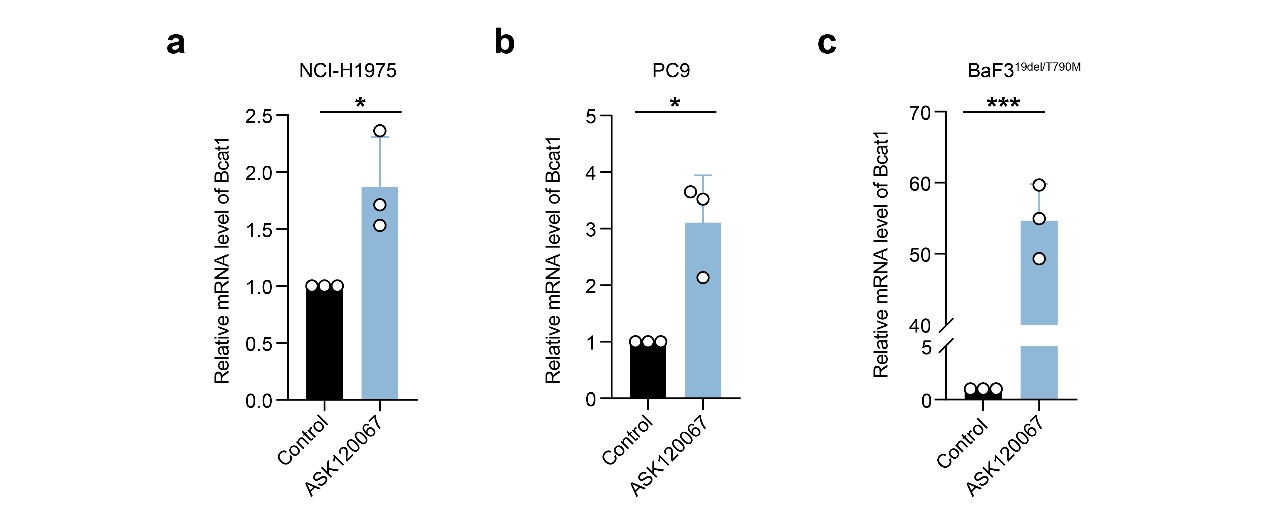
**

**Figure. S2.**

**Short-term treatment with ASK120067 induced BCAT1 upregulation in TKI-sensitive EGFR mutant cells.** **a, b, c** Changes of BCAT1 mRNA levels upon 1 μM ASK120067 treatment in NCI-H1975^L858R/T790M^ (**a**), and 200 nM ASK120067 treatment in PC9^19del^ (**b**) and EGFR^19del/T790M^-overexpressing BaF3 (**c**) cells for 3 days were determined by RT-qPCR (n=3). **p <* 0.05; ****p <* 0.001. Data are expressed as the mean ± SD.


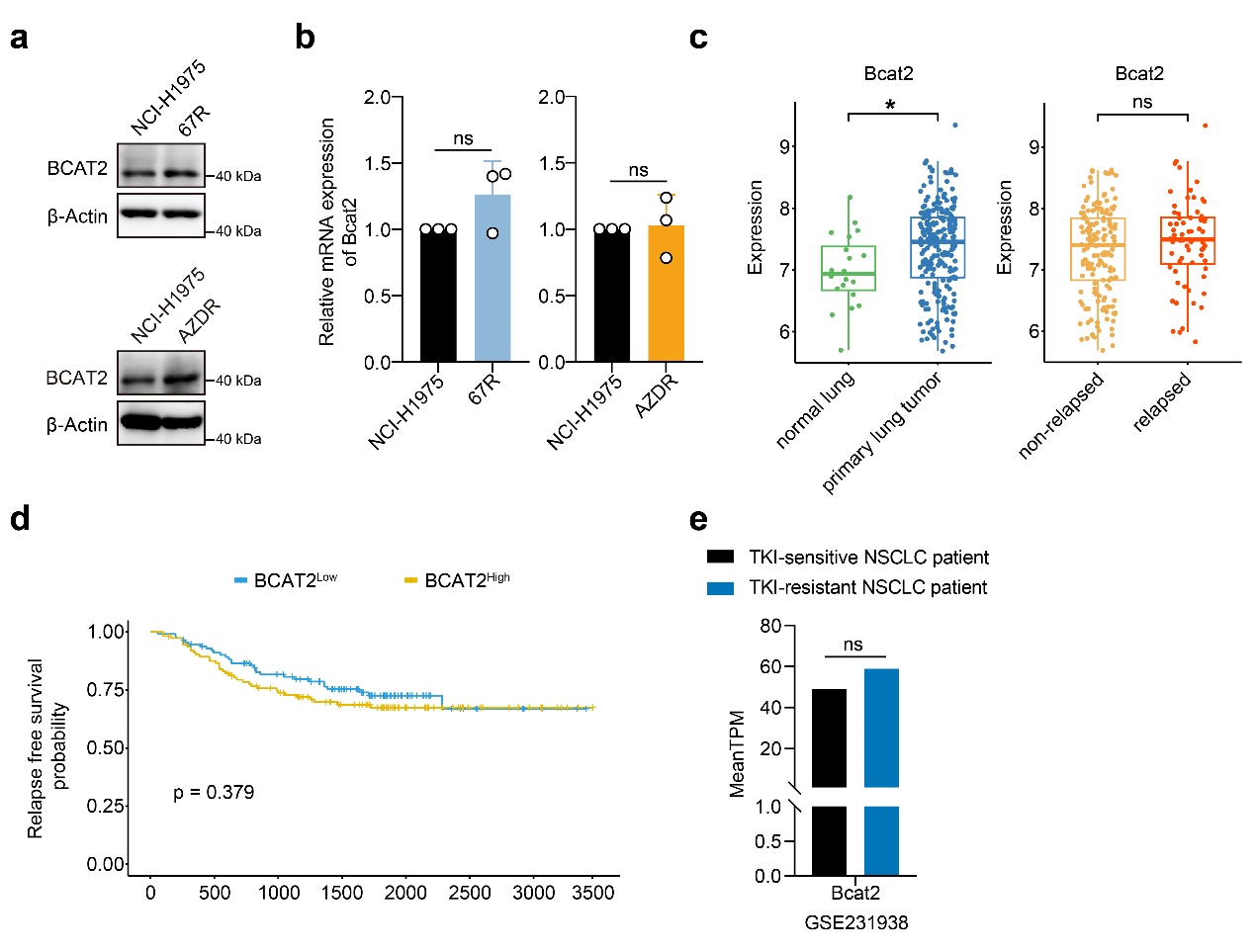


**Figure. S3.**

**Expression of BCAT2 in TKI-resistant cells compared to NCI-H1975 cells and its clinical implications.** **a, b** BCAT2 protein levels and relative mRNA levels in NCI-H1975 cells, ASK120067-resistant NCI-H1975 cells (67R), and osimertinib-resistant NCI-H1975 cells (AZDR) were assessed by Western blot assay (**a**) and RT- qPCR assay (**b**) (n=3). **c** Comparison of BCAT2 gene expression in normal lung tissue and primary lung adenocarcinoma tumors (left) or in tumor tissues from non-relapsed and relapsed lung adenocarcinoma patients (right) in the GSE31210 dataset. **d** Relapse-free survival probability of two groups of lung adenocarcinoma patients classified by BCAT2 median expression levels in the GSE31210 dataset. **e** Comparison of BCAT2 gene expression in tumors from TKI-sensitive- and TKI-resistant NSCLC patients in the GSE231938 dataset **p <* 0.05; ns, not significant. Data are expressed as the mean ± SD.


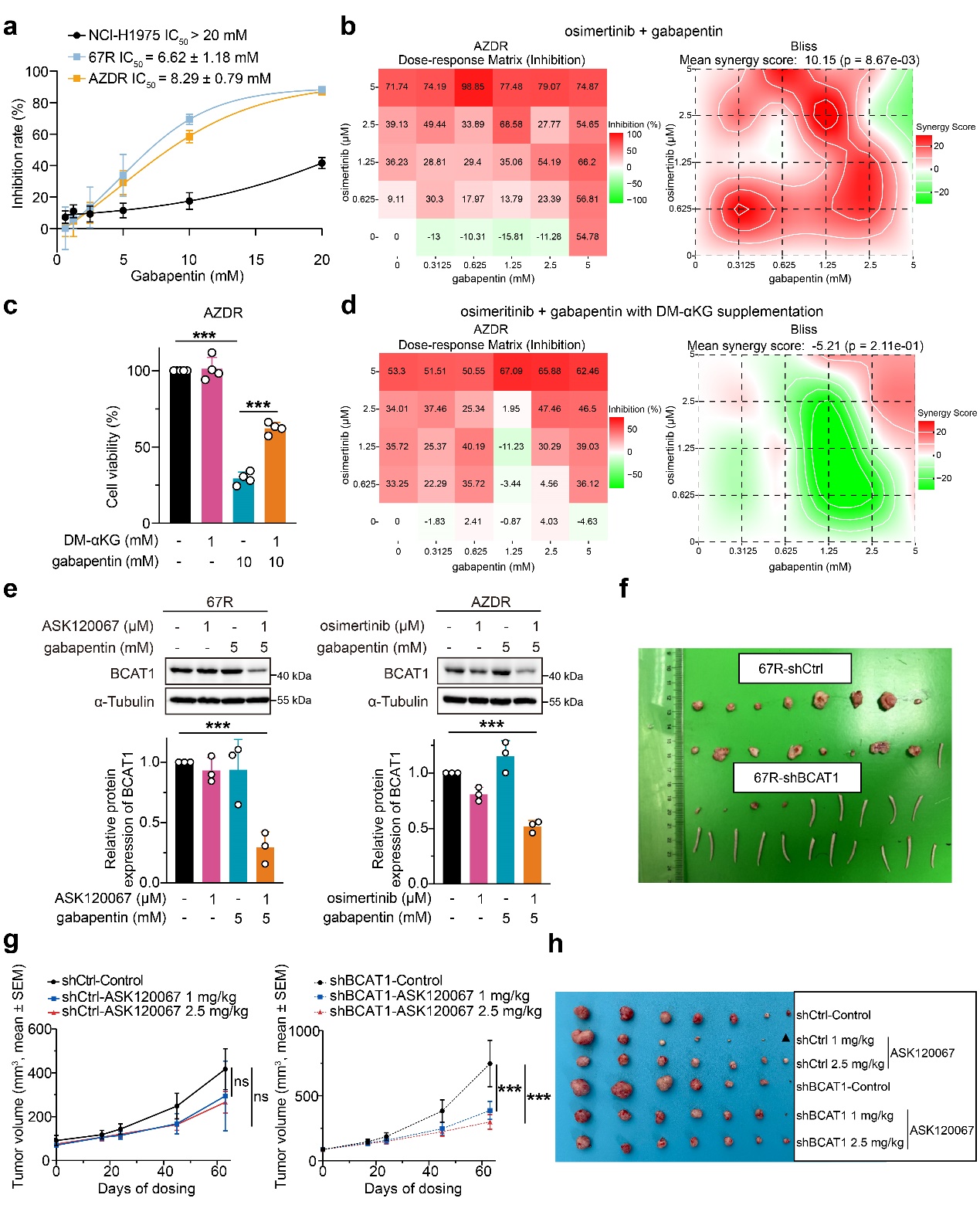


Figure. S4.

**BCAT1 inhibition restrained tumor growth and sensitized resistant tumors to EGFR TKI in an α-KG-dependent manner.** **a** Growth-inhibition activities of gabapentin in ASK120067-resistant NCI-H1975 cells (67R), osimertinib-resistant NCI-H1975 cells (AZDR) and NCI-H1975 cells were evaluated using Sulforhodamine B assay. **b** Effects of gabapentin in combination with EGFR TKI in AZDR were assessed using Sulforhodamine B assay and showed as dose-response matrix and synergy score matrix. **c** Cell viability of AZDR cells upon gabapentin, dimethyl-KG (DM-αKG), or combination treatment was evaluated using SRB colorimetric assay (n=4). **d** Anti-proliferation effects of gabapentin and osimertinib with DM-αKG supplementation on AZDR cells were detected using SRB colorimetric assay and showed as dose-response matrix and synergy score matrix. Synergy scores showed above were calculated by SynergyFinder using Bliss model. **e** BCAT1 expression levels upon gabapentin and EGFR TKI combination therapy were determined by Western blot assay and are shown as representative images (top) and quantitative graphs (bottom) (n=3). **f** Photographs of dissected tumors from BALB/c nude mice grafted with equal amount (10^7^) of control 67R (shCtrl) and BCAT1-knockdown 67R (shBCAT1) cells after 58 days. Mouse tails marked the mouse without tumor formation till the endpoint. **g** Tumor growth curve of ASK120067 (oral administration, 1 mg/kg or 2.5 mg/kg, once daily) for 63 days in control 67R (shCtrl) and BCAT1-knockdown 67R (shBCAT1) tumor models were evaluated. **h** Photo of dissected tumors at endpoint of 77 days were shown. “▲” marks the death of this mouse on 70^th^ day (n=7). ****p* < 0.001. Tumor volume data presented in (**g**) are shown as mean ± SEM, while other data are expressed as mean ± SD.


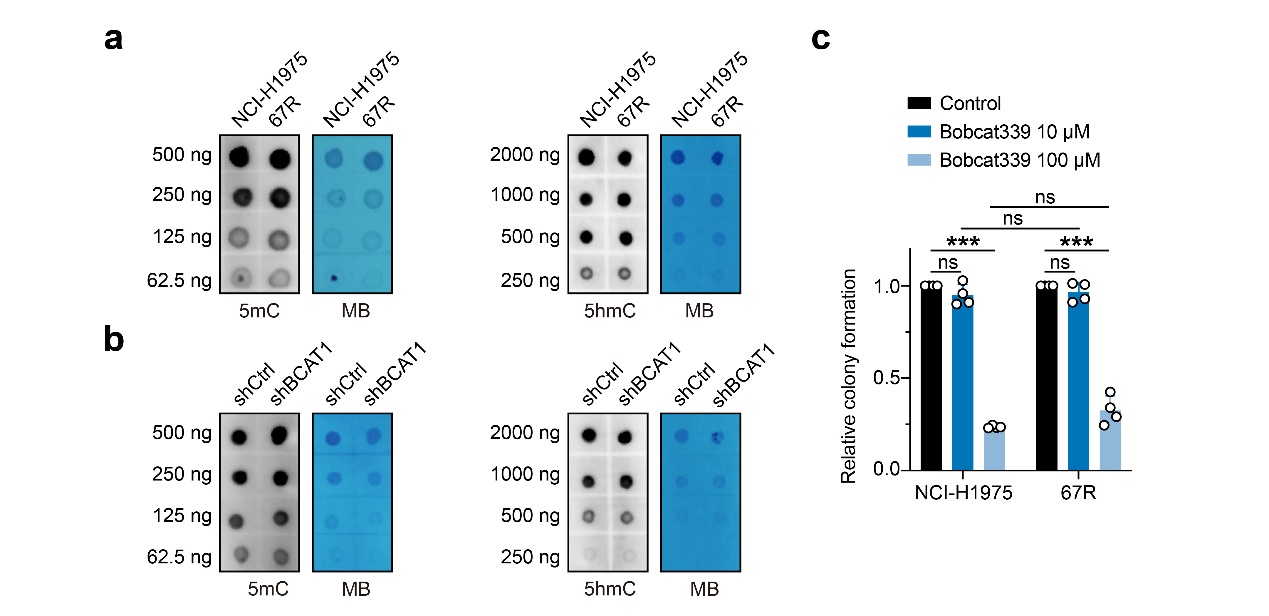


Figure. S5.

**TET-dependent DNA demethylation might not be involved in BCAT1-mediated resistance to EGFR-TKIs.** **a, b** Dot blot analysis of 5mC and 5hmC levels of genomic DNA between NCI-H1975 and 67R cells (**a**) and between control 67R cells (shCtrl) and BCAT1-knockdown 67R cells (shBCAT1) (**b**). **c** Colony formation assay was performed to examine the anti-growth effects of Bobcat339 on NCI-H1975 or 67R cells (n=4). ****p <* 0.001; ns, not significant. Data are expressed as the mean ± SD.


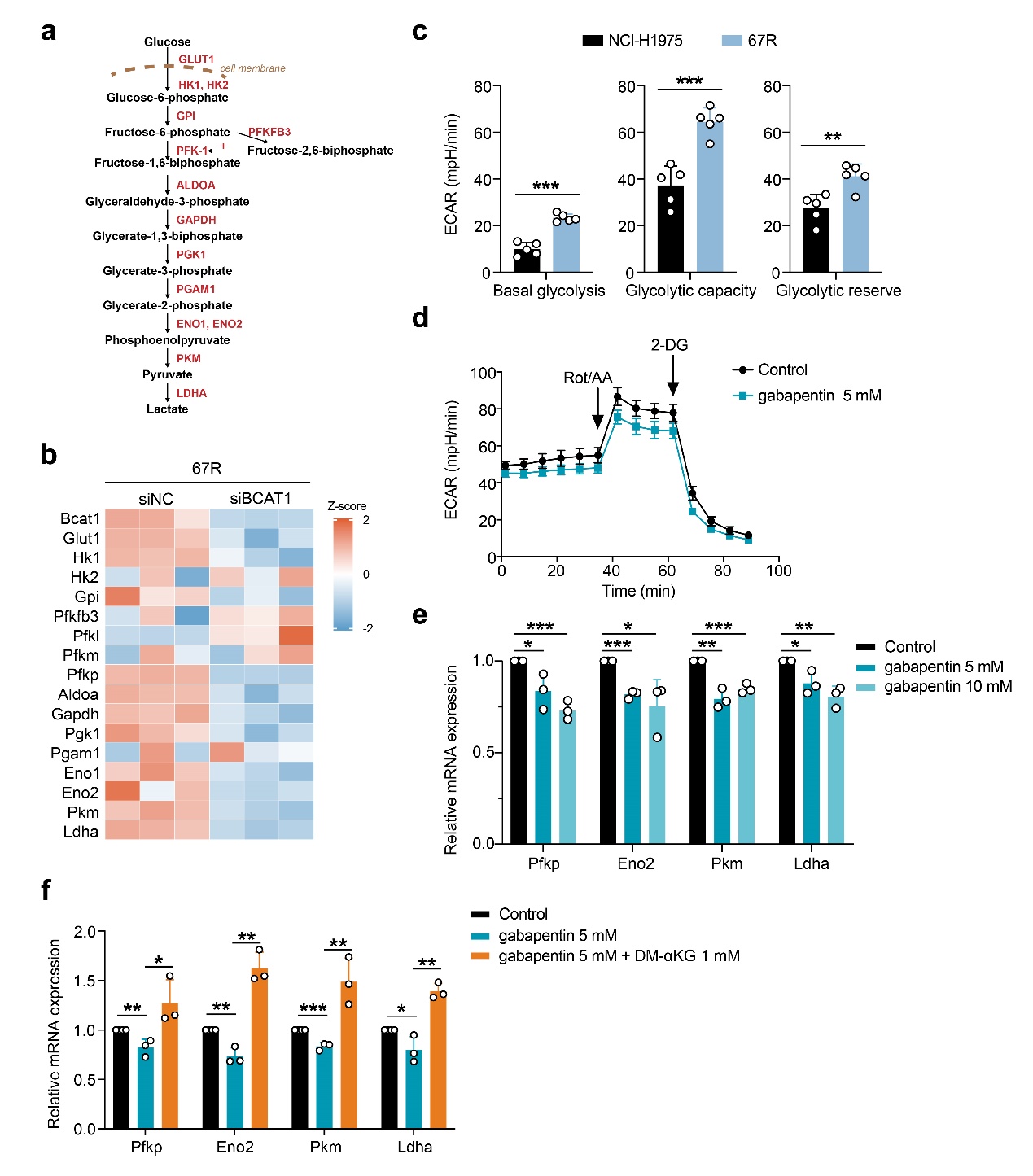


Figure. S6.

**BCAT1 promoted glycolysis in 67R through αKG-dependent upregulation of glycolysis-related enzymes.** **a** Schematic of key enzymes related to the glycolysis pathway. **b** Relative expression levels of glycolysis-related enzymes in control 67R cells (siNC) and BCAT1-knockdown 67R cells (siBCAT1) were determined by RNA-seq and normalized mRNA abundances were shown as a heatmap. **c** ECAR analysis of NCI-H1975 cells and 67R cells and shown as quantitative graphs (n=5). **d** ECAR analysis of 67R cells treated with 5 mM gabapentin for 3 days. Rot/AA: rotenone and antimycin A; 2-DG: 2-Deoxy- d-glucose. **e** RT-qPCR assay for glycolysis genes in 67R cells treated with PBS control or gabapentin for 3 days (n=3). **f** RT-qPCR assay for glycolysis genes in 67R cells upon gabapentin treatment with or without α-KG supplementation. Cells were treated with PBS or gabapentin for 3 days with or without DM-αKG for 24 h (n=3). **p <*0.05, ***p <* 0.01, ****p <* 0.001. Data are expressed as the mean ± SD.


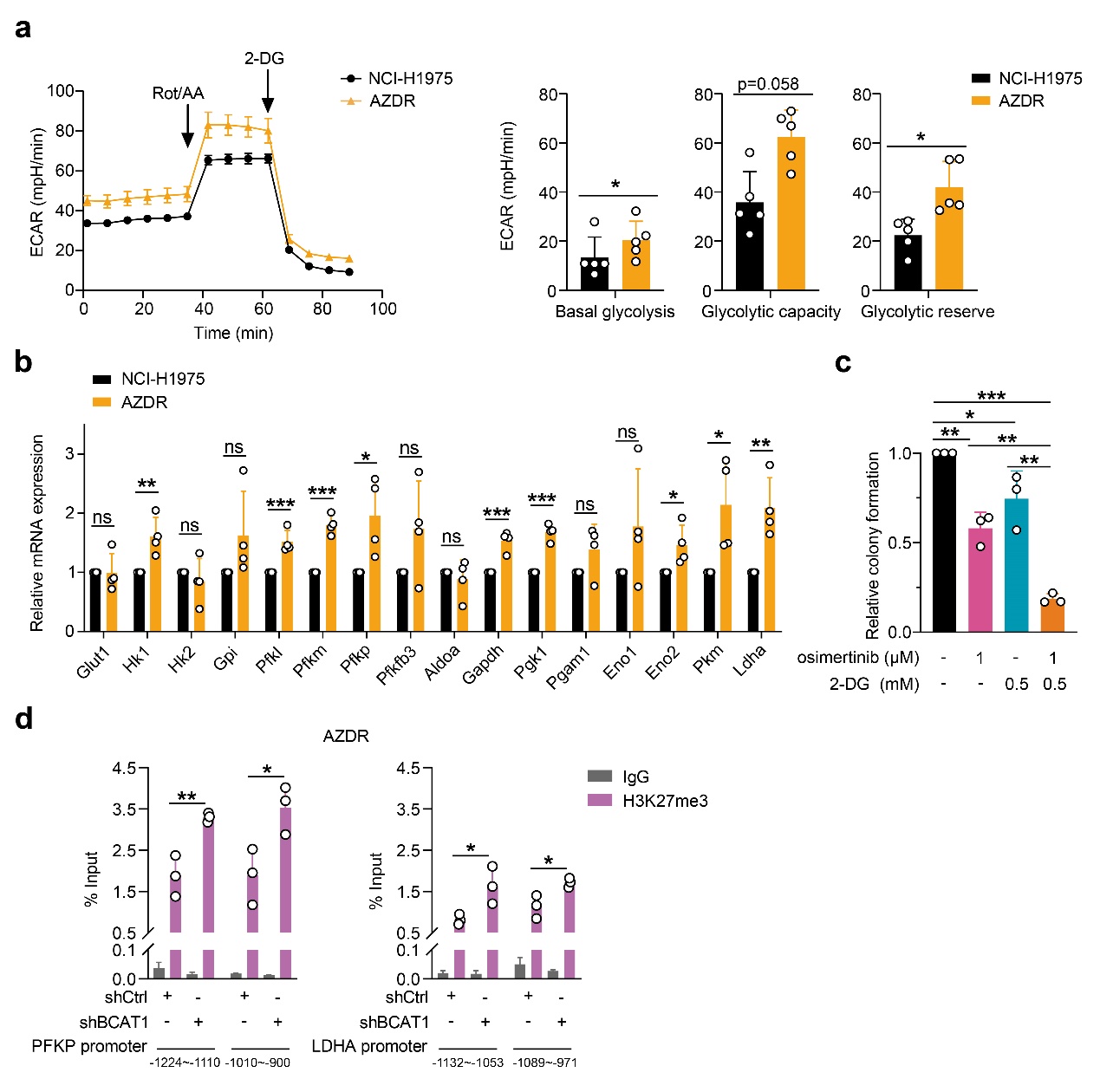


Figure. S7.

**BCAT1 enhanced glycolysis in AZDR through transcriptional derepressing of glycolysis-related genes**. **a** Extracellular acidification rates (ECARs) of NCI-H1975 and AZDR cells were detected using a Seahorse XF Analyzer and shown as representative time-dependent curve (left) and quantitative graphs (right) (n=5). Rot/AA: rotenone and antimycin A; 2-DG: 2-Deoxy-d-glucose. **b** Relative mRNA levels of glycolytic enzymes in NCI-H1975 and AZDR cells were evaluated by RT-qPCR assay (n=4). **c** Quantification of relative colony formation of AZDR cells upon osimertinib, 2-DG or combination therapy (n=3). **d** ChIP-qPCR analysis of H3K27me3 abundance at the promoters of PFKP (p1, -1224~-1110; p2, -1010~-900) and LDHA (p1, -1132~-1053; p2, -1089~-971) in AZDR shCtrl and AZDR shBCAT1 cells (n=3). **p <*0.05, ***p <* 0.01, ****p <* 0.001, ns, not significant. Data are expressed as the mean ± SD.


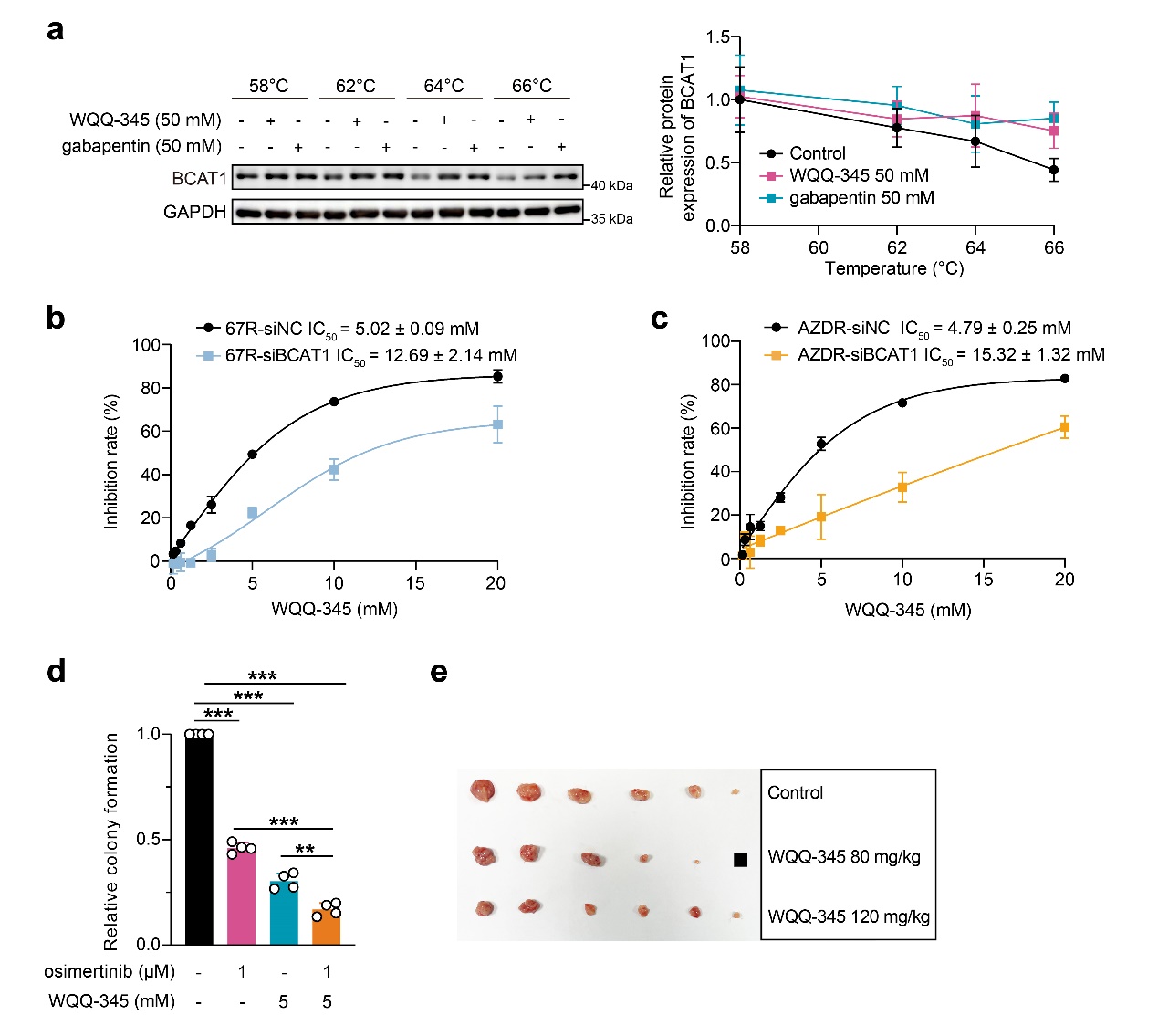


Figure. S8.

**Efficacy of WQQ-345 on TKI-resistant cells and tumor as a BCAT1 inhibitor. a** A thermal shift assay was conducted to measure the thermal stability of cellular BCAT1 in 67R cells pre-incubated with or without gabapentin or WQQ-345. **b, c** Anti-growth activities of WQQ-345 in ASK120067-resistant NCI-H1975 cells (67R) (**b**) and osimertinib-resistant NCI-H1975 cells (AZDR) (**c**) with or without BCAT1 knockdown were examined using Sulforhodamine B assay. **d** Quantification of relative colony formation of AZDR cells treated with vehicle control, osimertinib, WQQ-345 and drug combination (n=4), normalized against the control. **e** The *in vivo* antitumor activity of WQQ-345 was evaluated in a 67R xenograft tumor model. 67R tumor-bearing mice were given oral treatments of PBS control or WQQ-345 twice daily for 43 days, and photo of dissected tumors at endpoint was shown. “■” marks no tumor dissected from this mouse. ***p* < 0.01, ****p* < 0.001; ns, not significant. Data are expressed as mean ± SD.


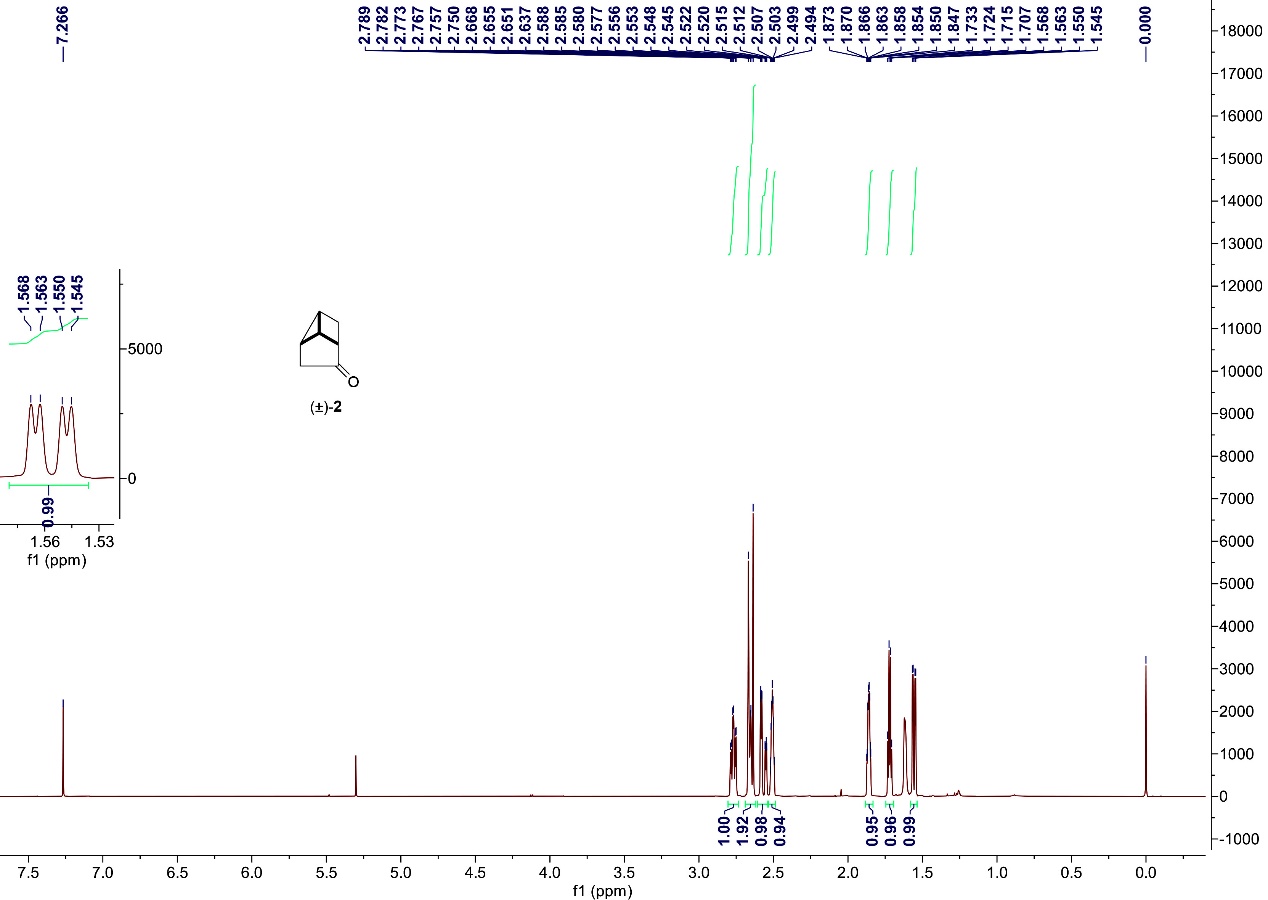


**Figure. S9.** ^1^H NMR spectrum of (±)-**2**


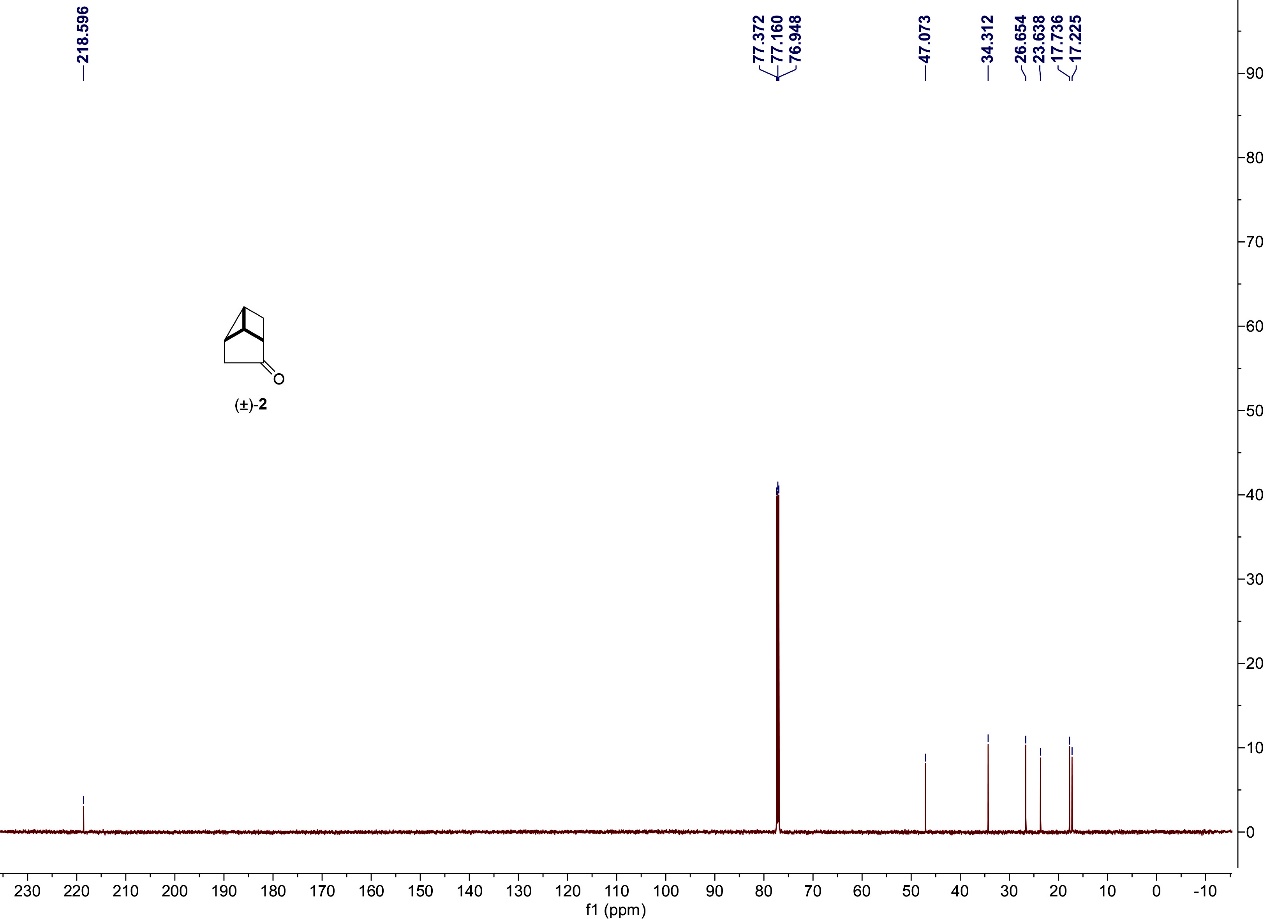


**Figure. S10.**^13^C NMR spectrum of (±)-**2**


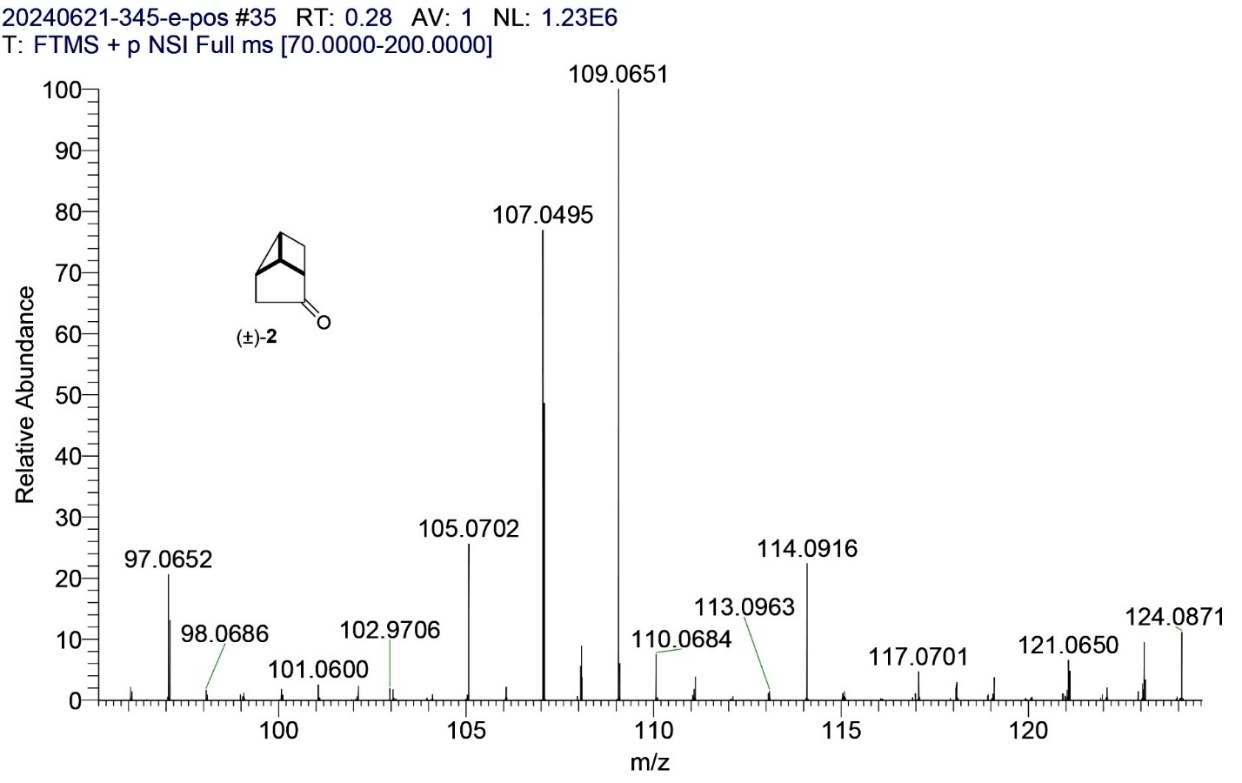


**Figure. S11.** HRMS spectrum of (±)-**2**


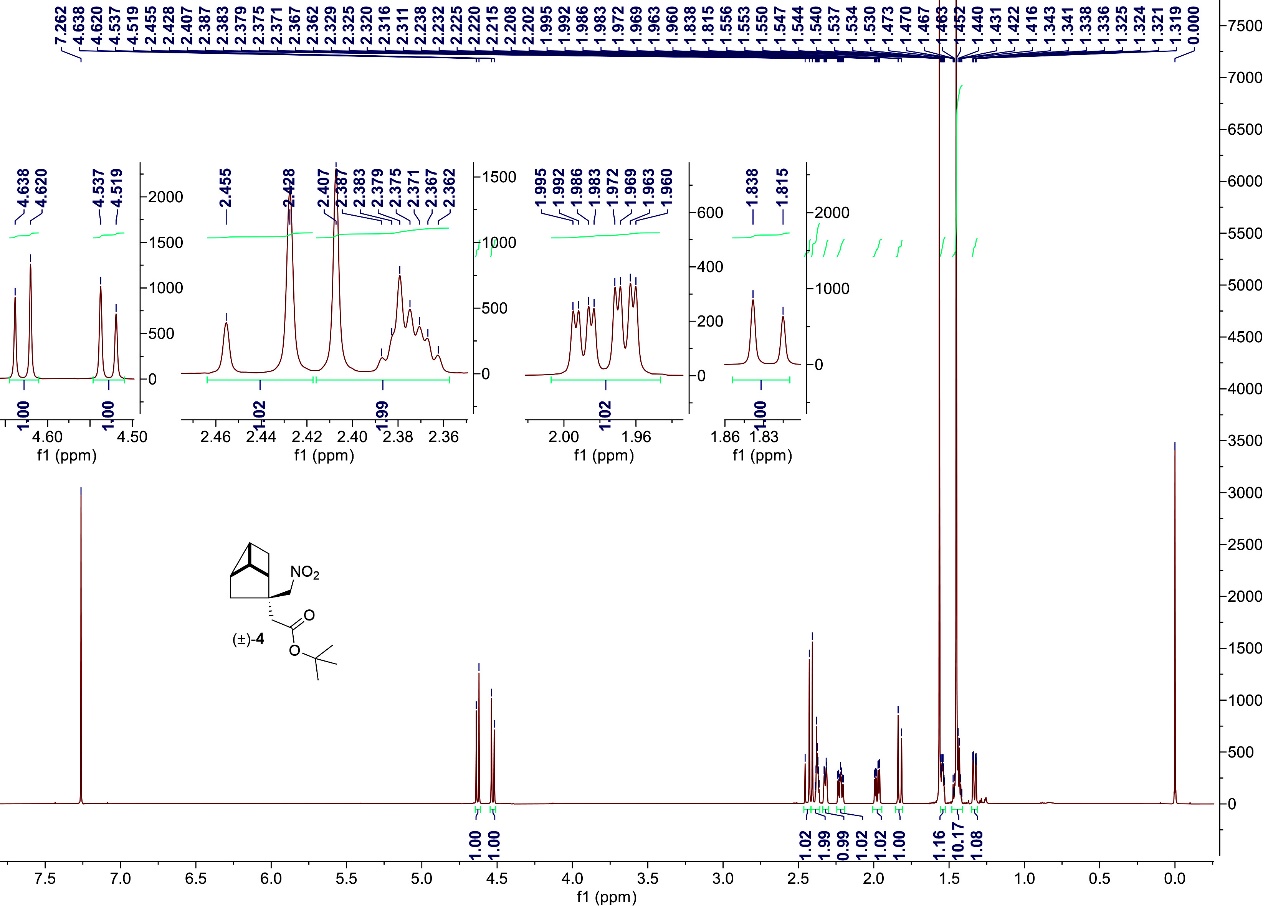


**Figure. S12.** ^1^H NMR spectrum of (±)-**4**


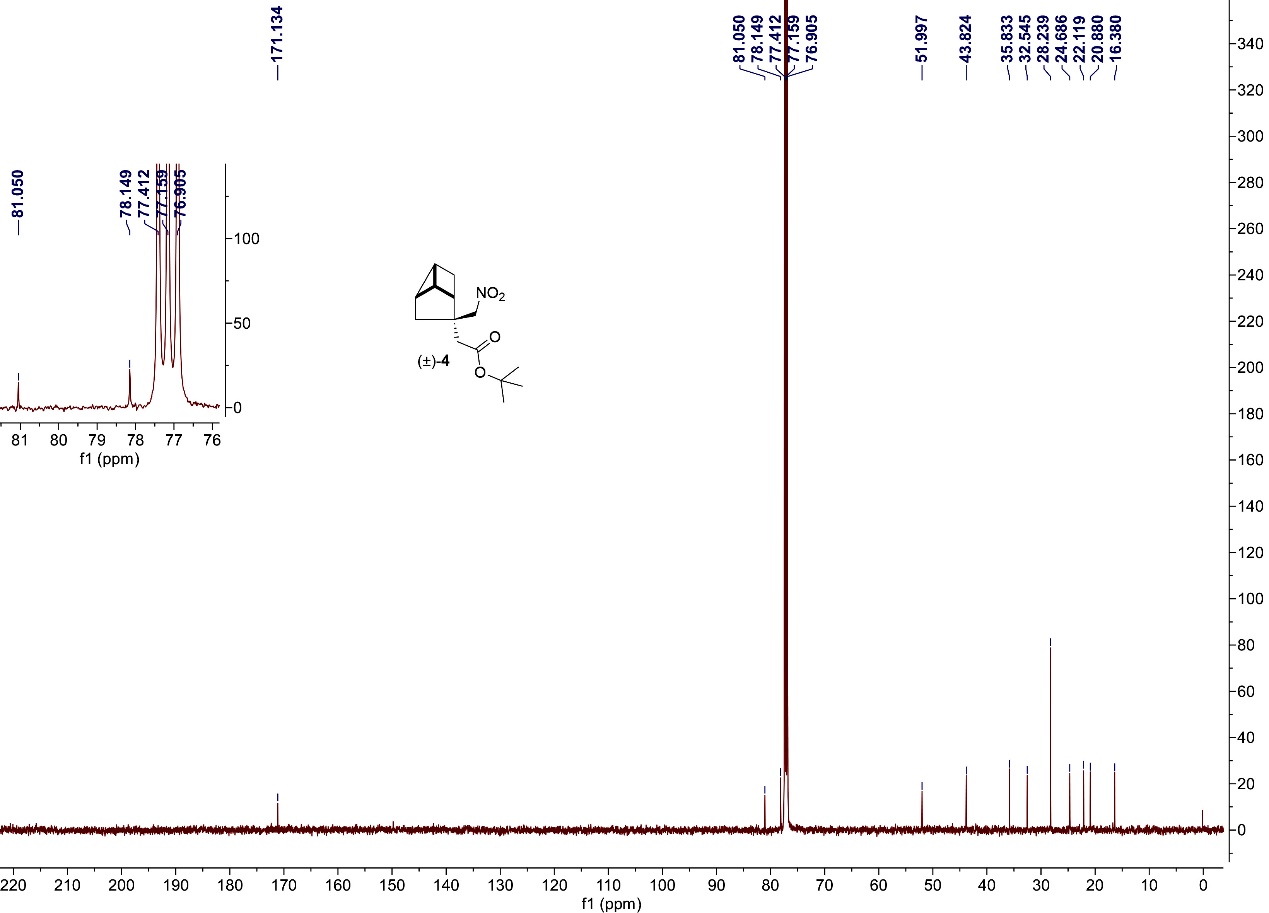


**Figure. S13.** ^13^C NMR spectrum of (±)-**4**


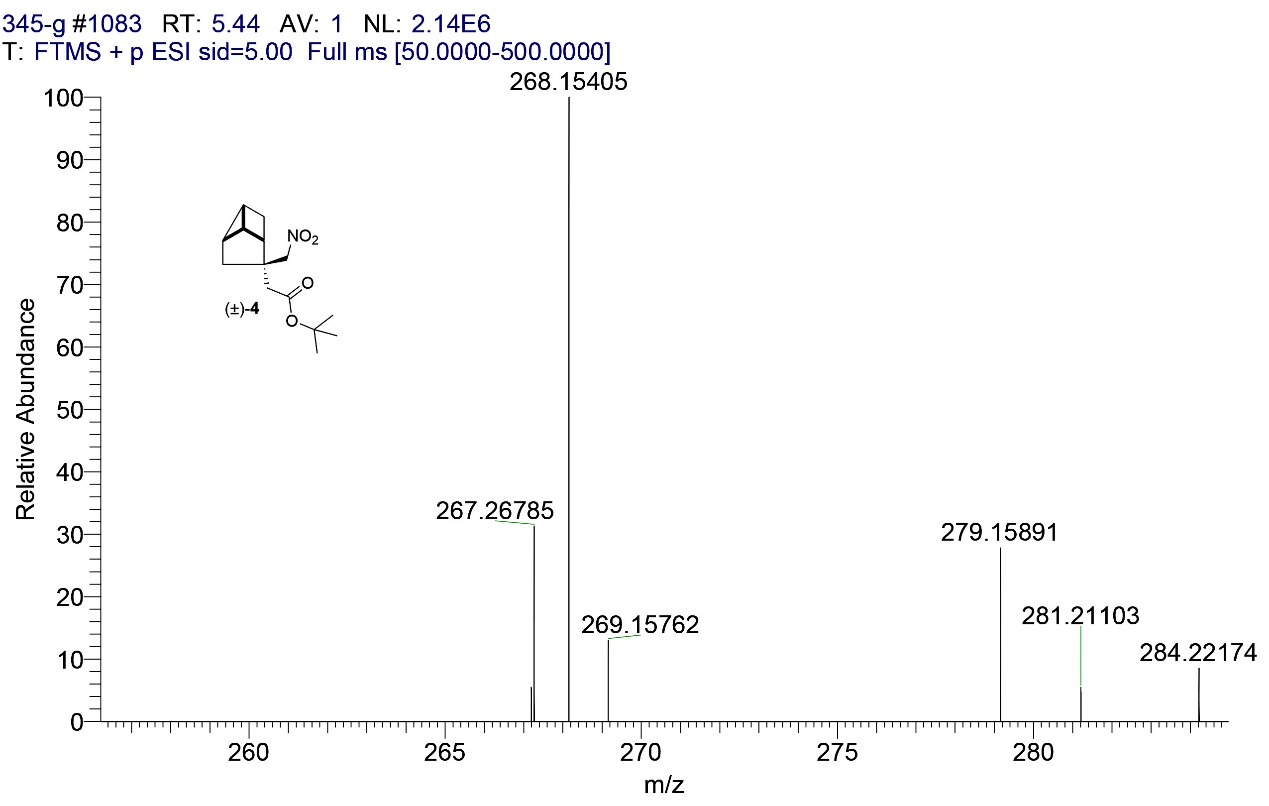


**Figure. S14.** HRMS spectrum of (±)-**4**


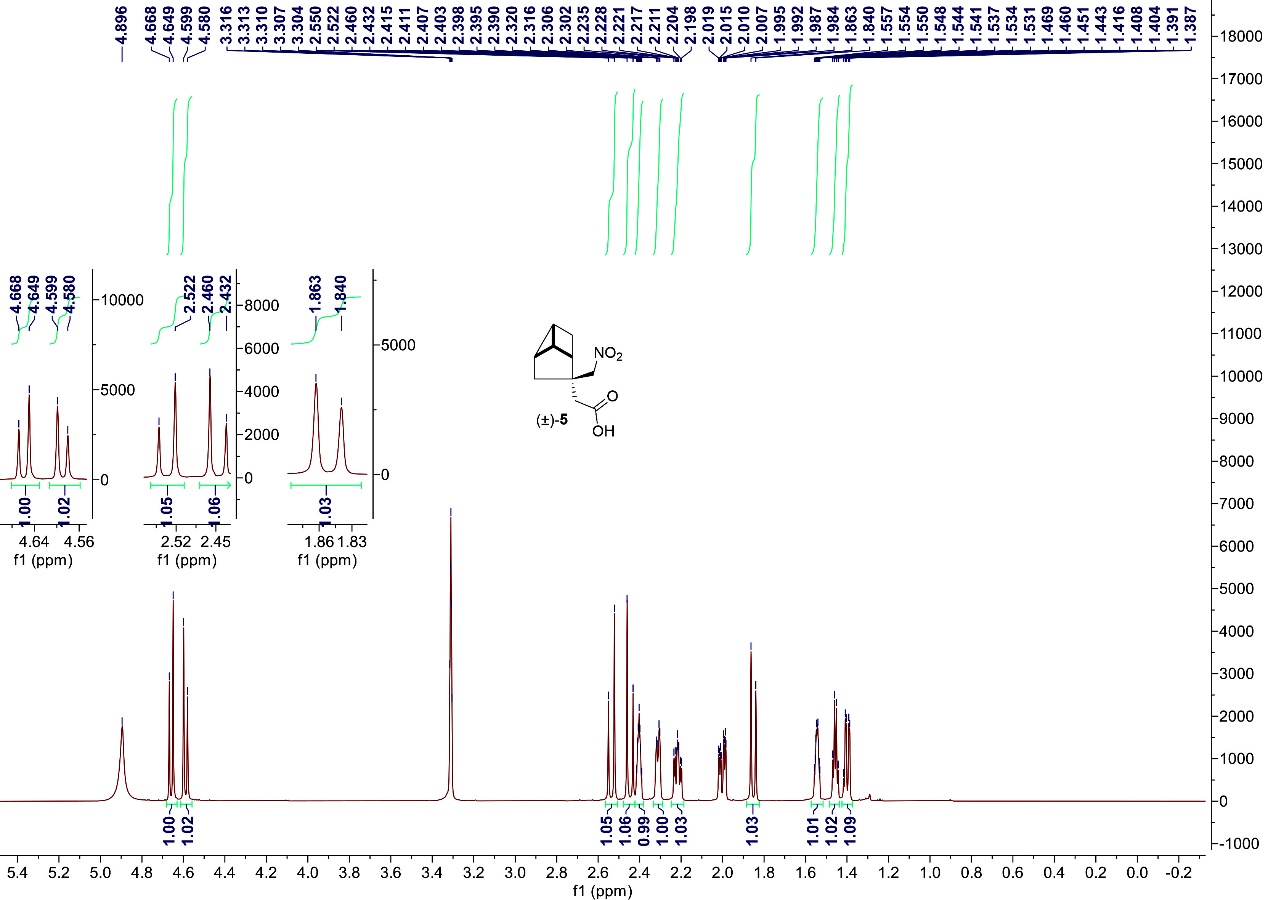


**Figure. S15.** ^1^H NMR spectrum of (±)-**5**


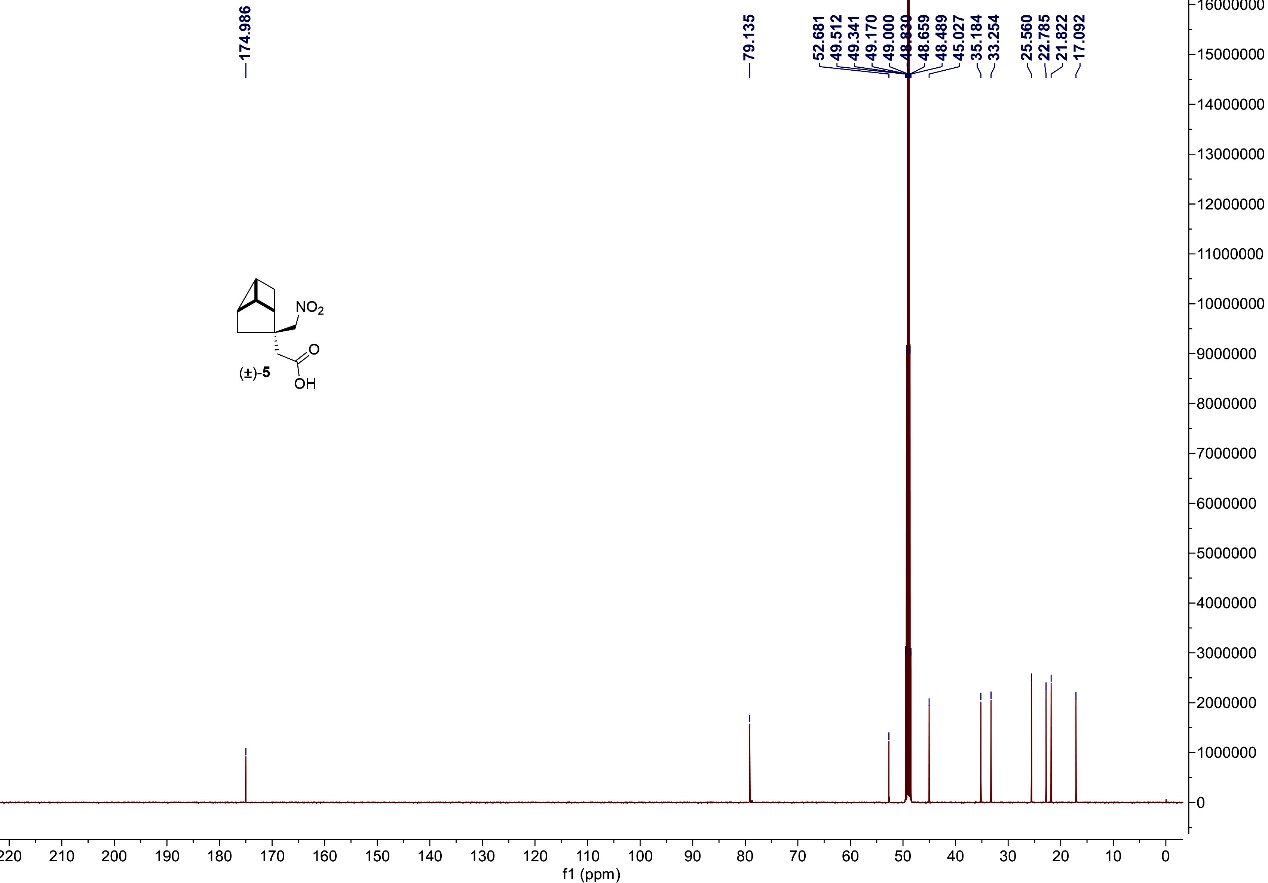


**Figure. S16.** ^13^C NMR spectrum of (±)-**5**


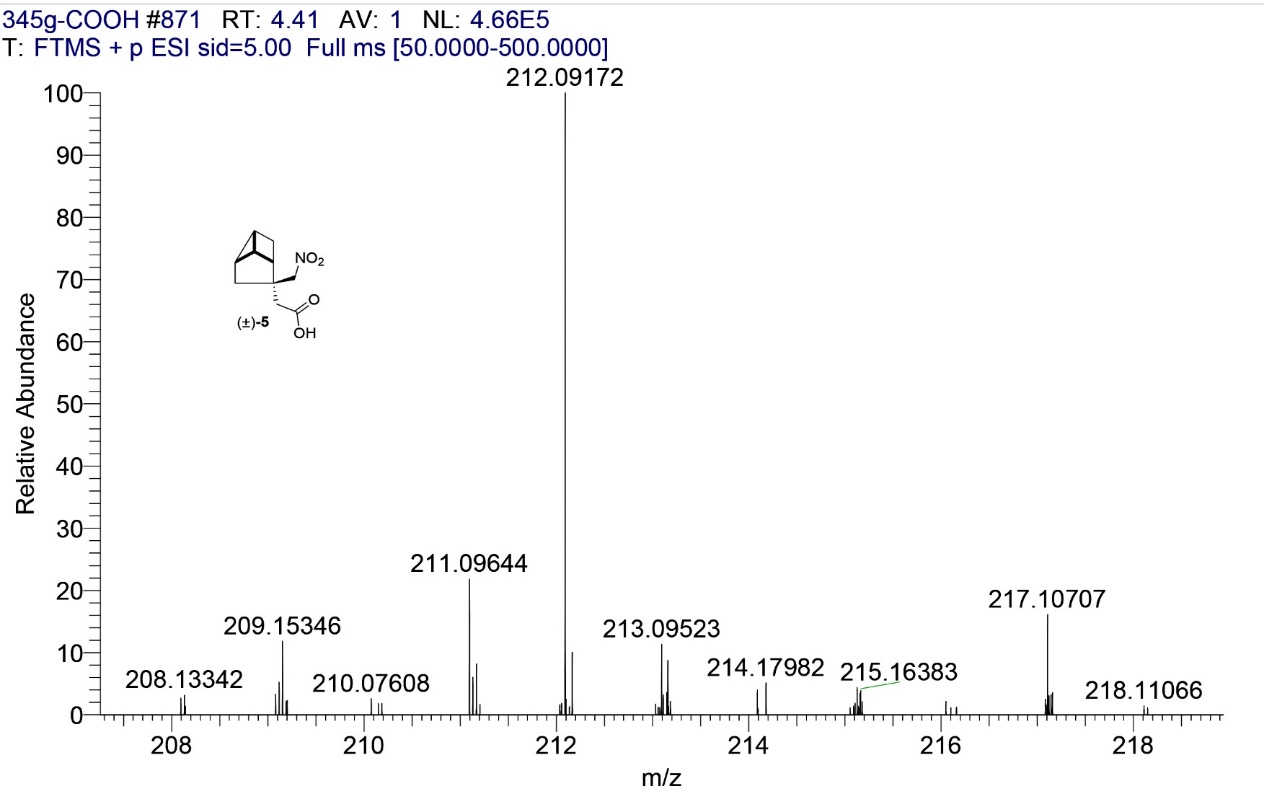


**Figure. S17.** HRMS spectrum of (±)-**5**


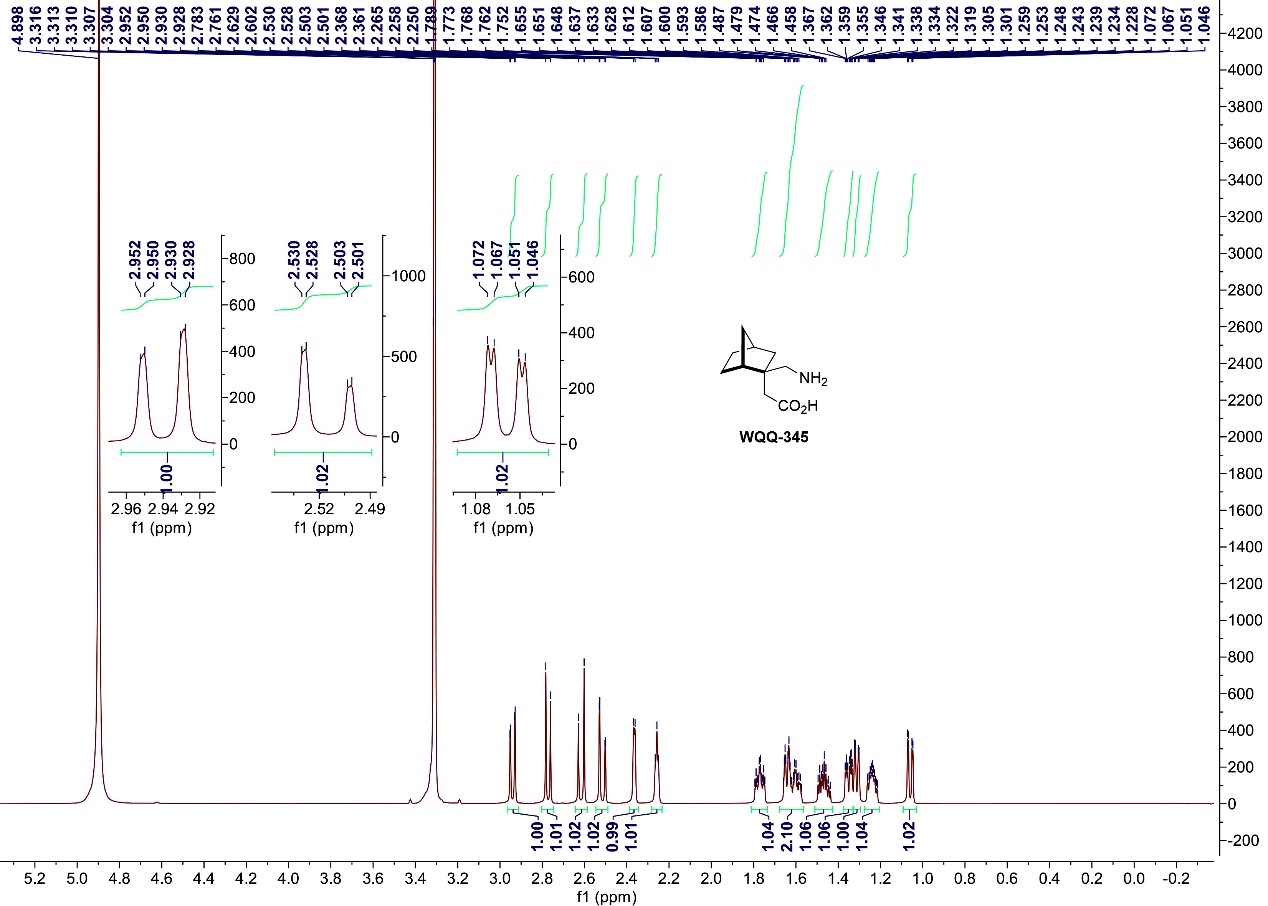


**Figure. S18.** ^1^H NMR spectrum of **WQQ-345**


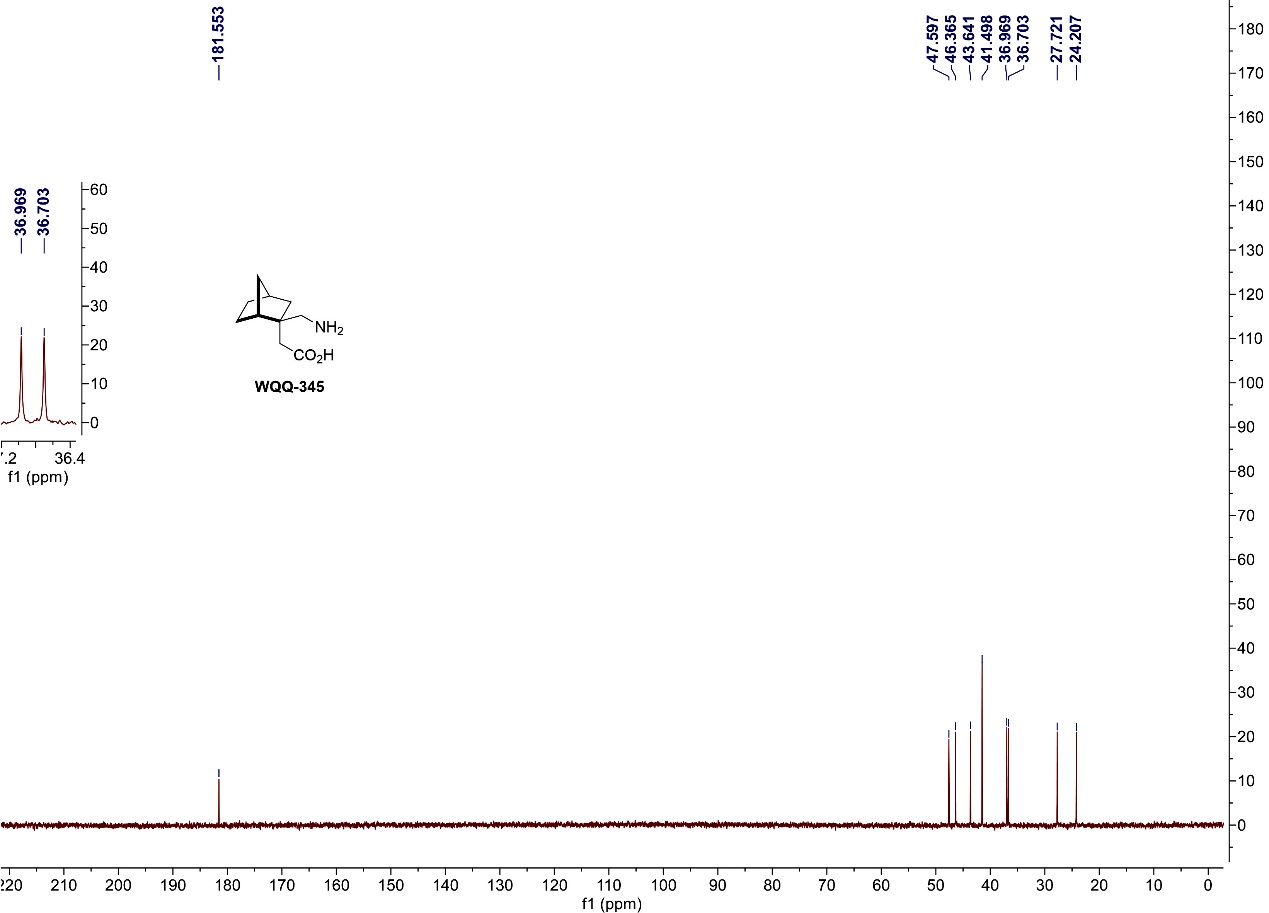


**Figure. S19.** ^13^CNMR spectrum of **WQQ-345**


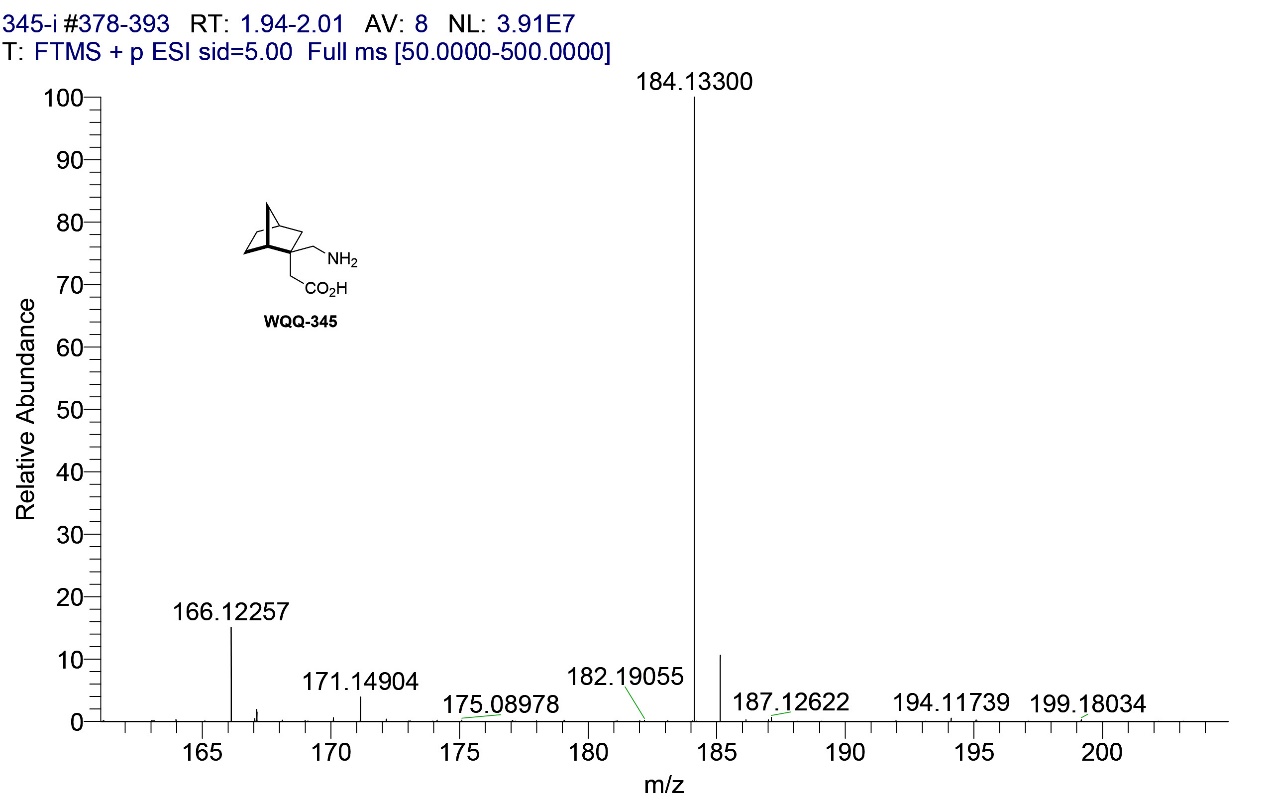


**Figure. S20.** HRMS spectrum of **WQQ-345**


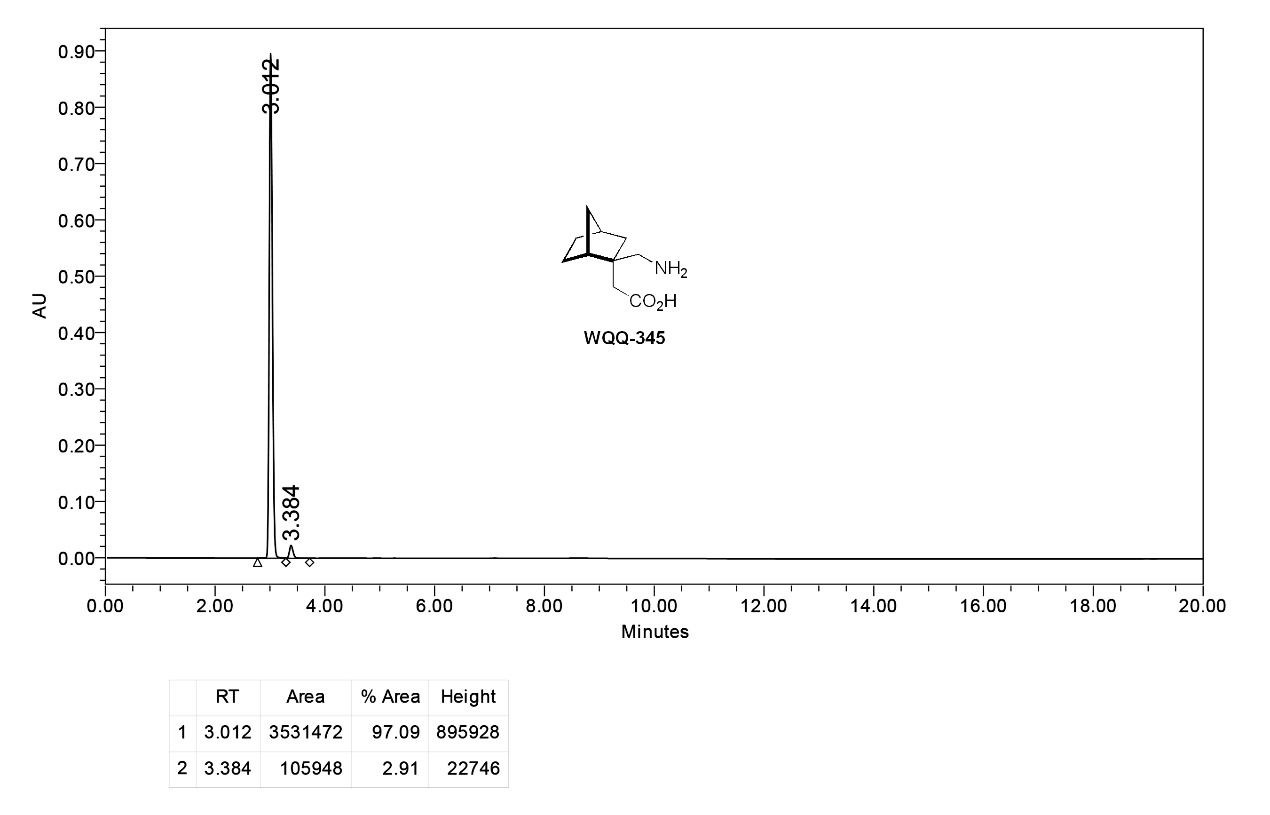


Figure. S21.

**HPLC chromatogram of WQQ-345.** HPLC condition: Analytical HPLC (Waters Arc); Column, C_18_, 4.6 × 150 mm, 3 μm (NanoChrom); Wavelength, 210 nm; Column temperature, 25°C; Flow rate, 0.6 mL/min. A “phosphate buffer” was prepared by dissolving 1.08 g KH_2_PO_4_ in 900 mL of pure water followed by adjustment of pH to 6-7 by 2.5 M aqueous KOH. Mobile phase A = “phosphate buffer”/MeCN (94/6), while mobile phase B = “phosphate buffer”/MeCN (70/30). Gradient elution, *t* = 0 min, mobile phase A/mobile phase B = 100/0, *t* = 8 min, mobile phase A/mobile phase B = 100/0, *t* = 15 min, mobile phase A/mobile phase B = 0/100, *t* = 20 min, mobile phase A/mobile phase B = 0/100.


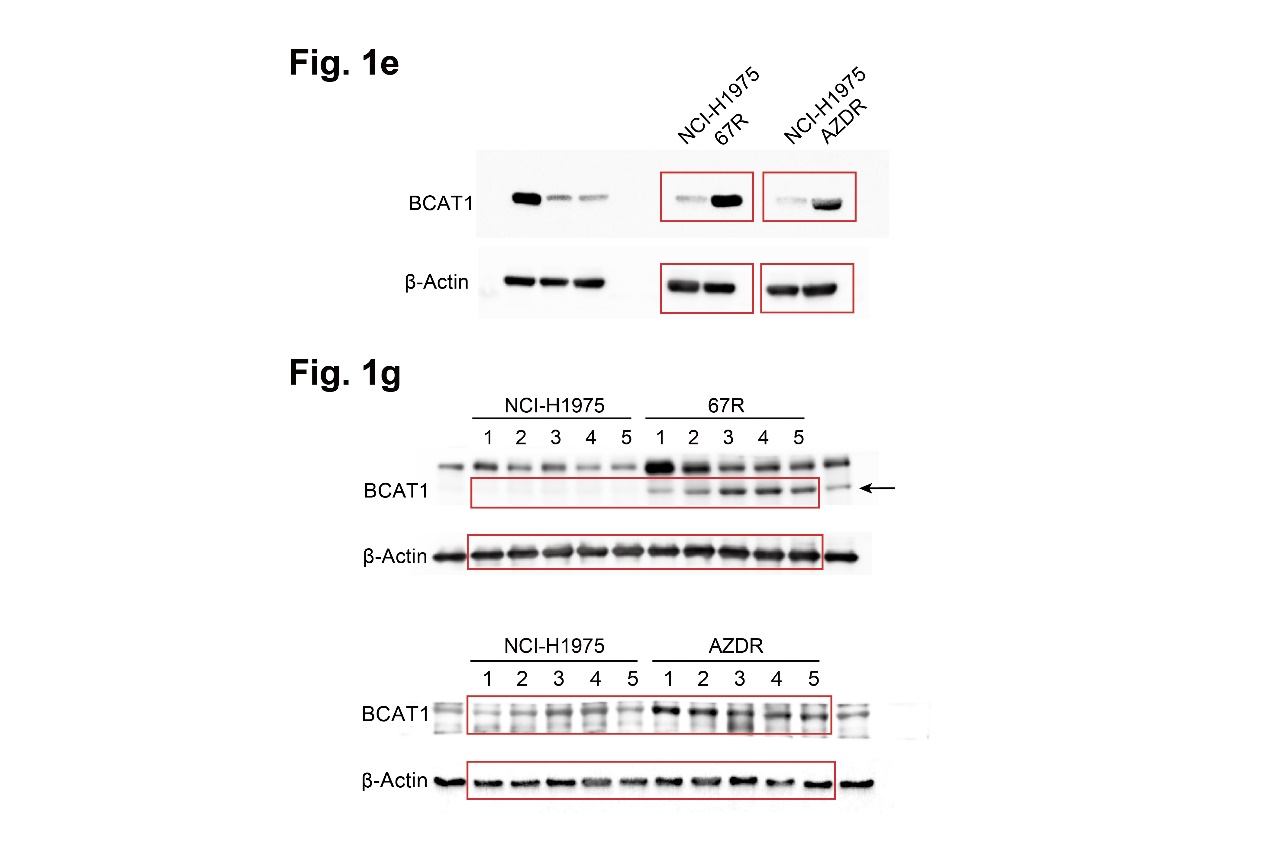


Figure. S22.

**Raw data of western blot related to Fig. 1e and 1g.**


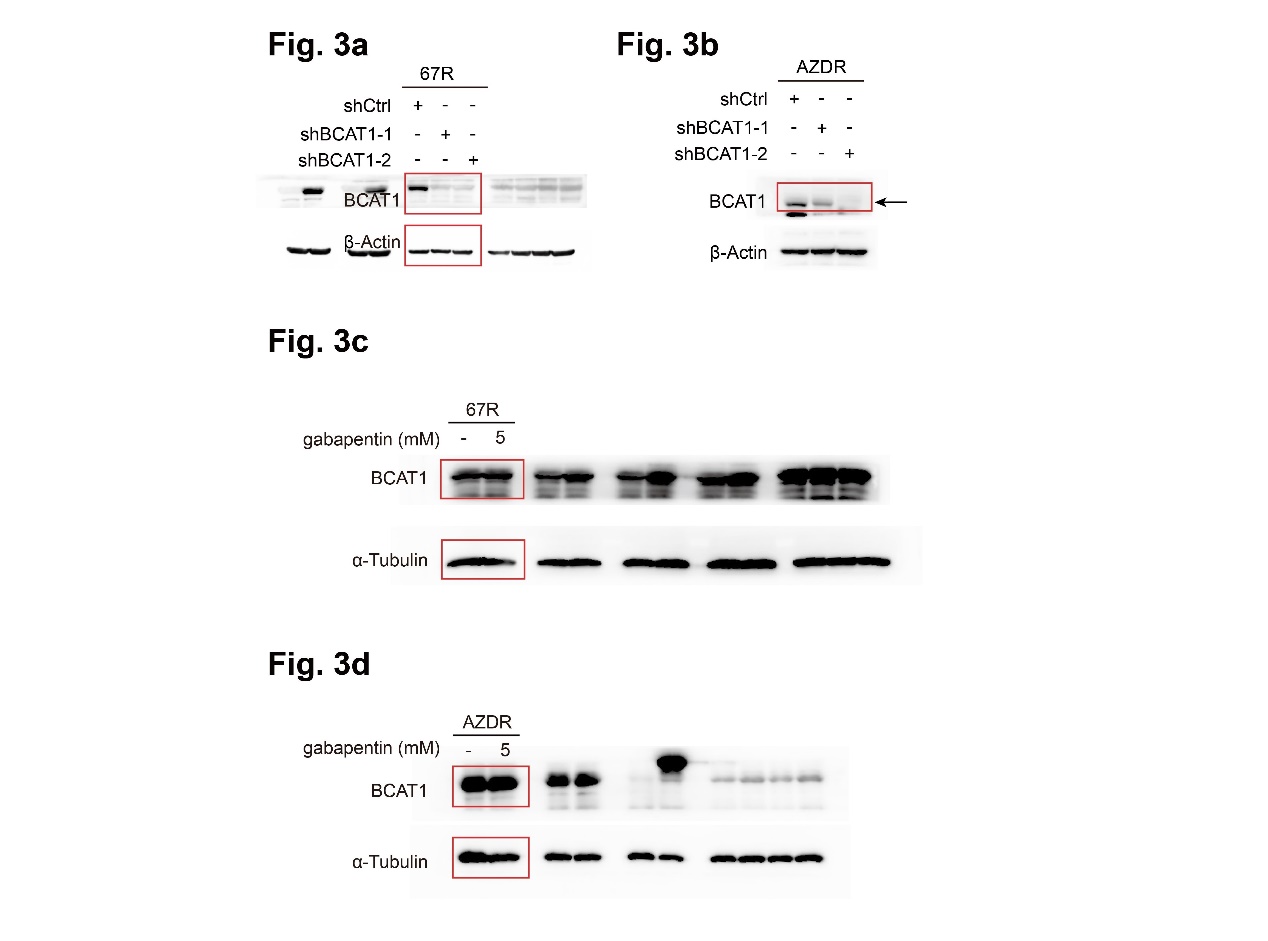


Figure. S23.

**Raw data of western blot related to Fig. 3a, 3b, 3c and 3d.**


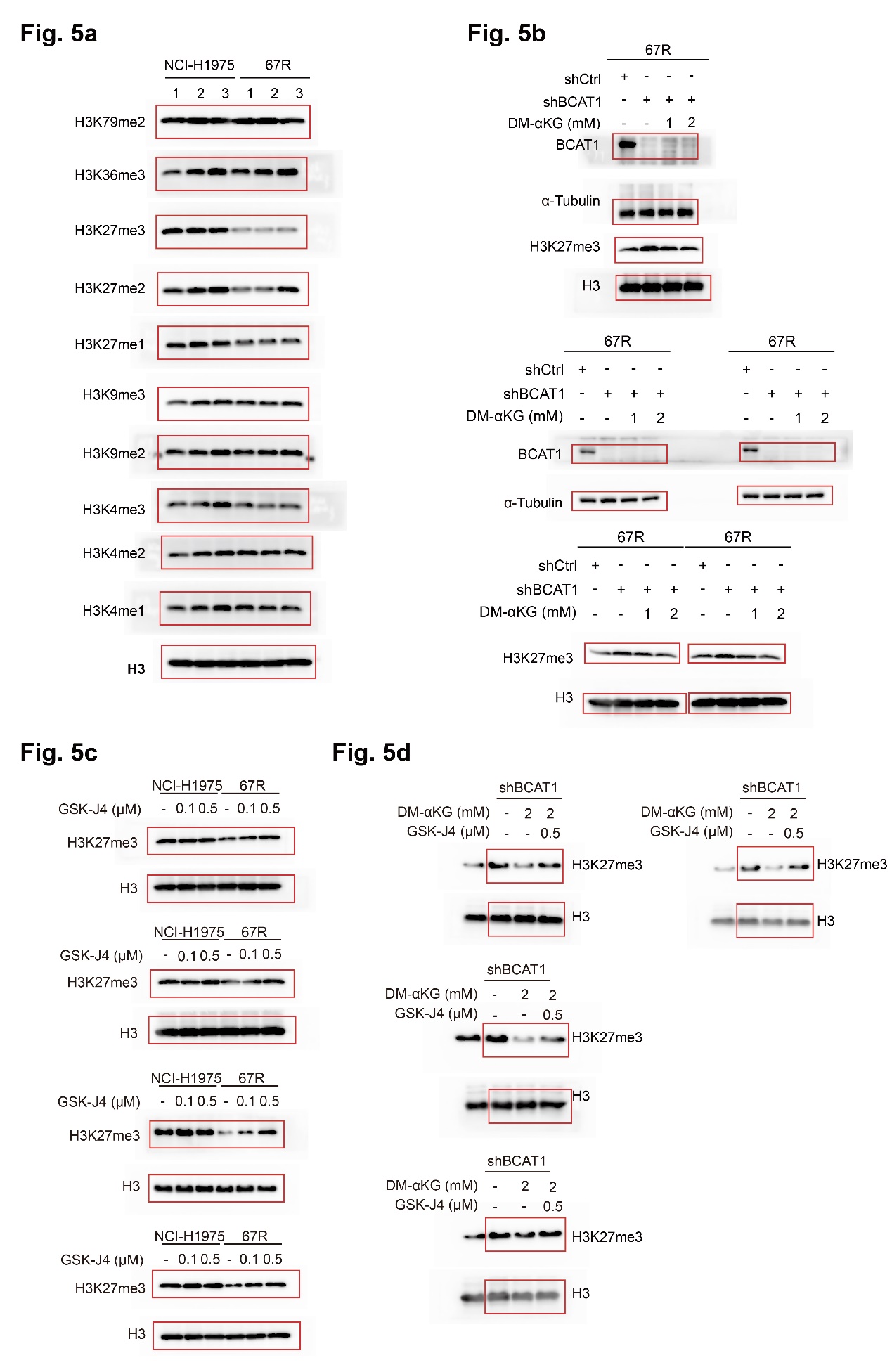


Figure. S24.

**Raw data of western blot related to Fig. 5a, 5b, 5c and 5d.**


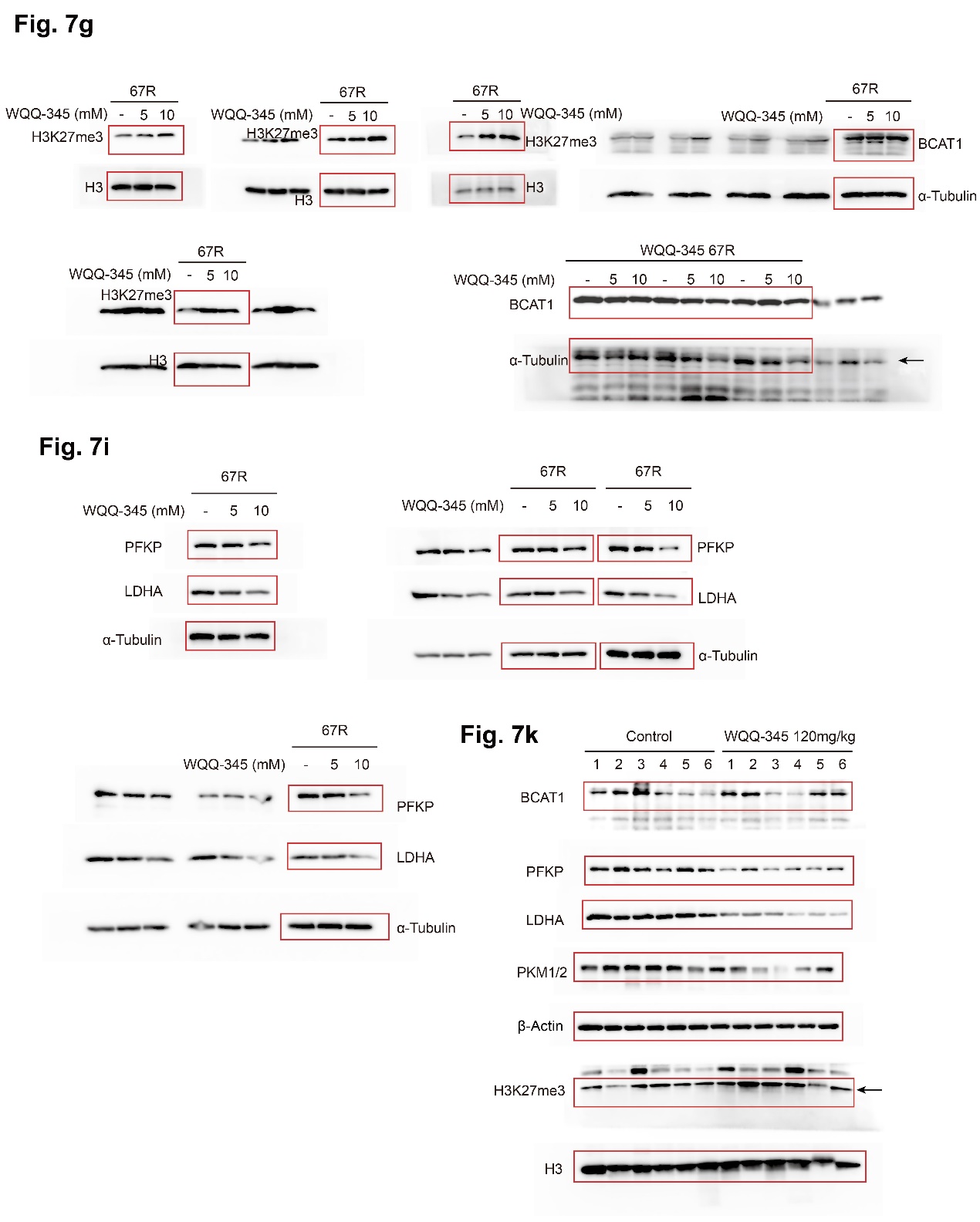


Figure. S25.

**Raw data of western blot related to Fig. 7g, 7i and 7k.**


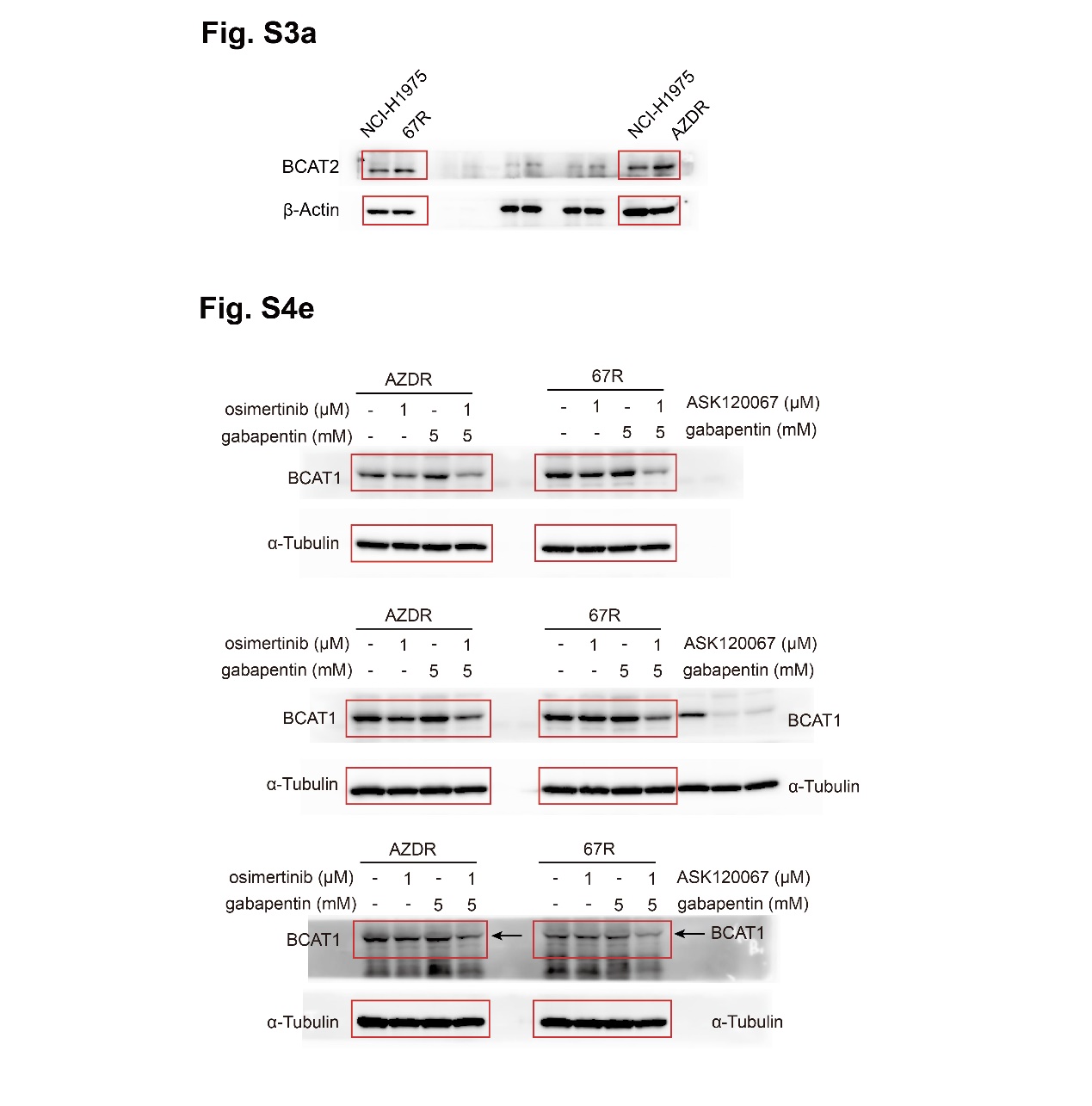


Figure. S26.

**Raw data of western blot related to Fig. S3a and Fig. S4e.**


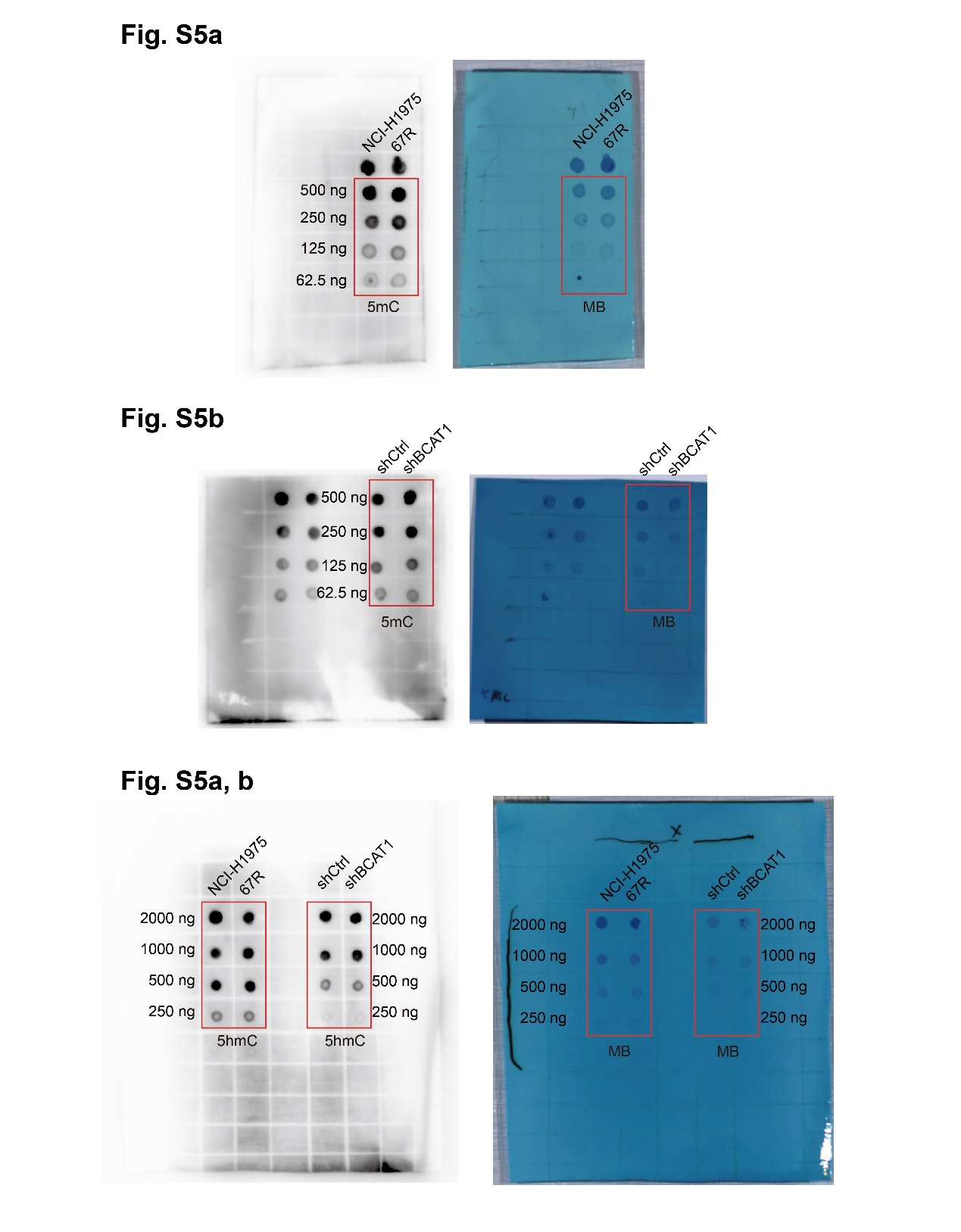


Figure. S27.

**Raw data of western blot related to Fig. S5a and Fig. S5b.**


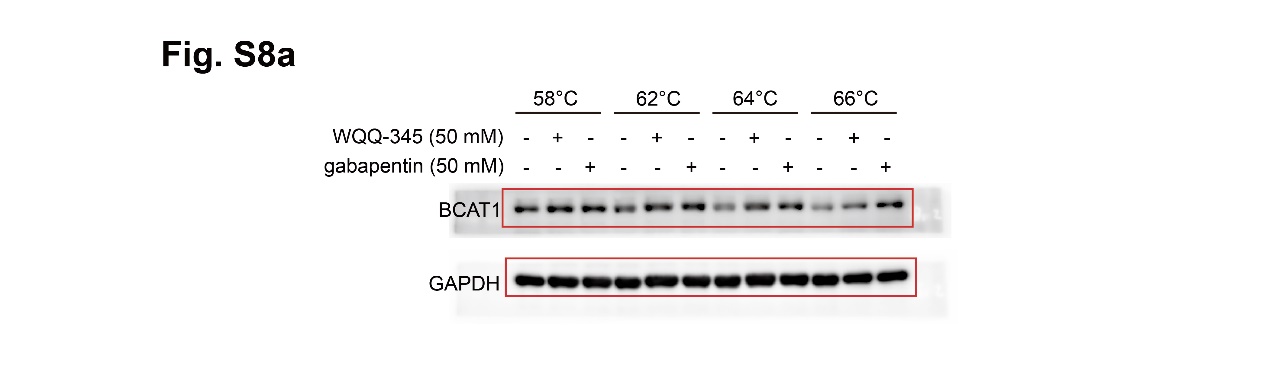


Figure. S28.

**Raw data of western blot related to Fig.S8a.**

Table S1.

**Sequences of RT-qPCR primers**

| **Primer** | **Sequence (5’ to 3’)** |
| --- | --- |
| Actin-F | CACCATTGGCAATGAGCGGTTC |
| Actin-R | AGGTCTTTGCGGATGTCCACGT |
| Aldoa-F | GCTGTCACTGGGATCACCTTC |
| Aldoa-R | GCTCGGAGTGTACTTTCCTTGA |
| Eno1-F | GACTTGGCTGGCAACTCTG |
| Eno1-R | GGTCATCGGGAGACTTGAA |
| Eno2-F | TCATGGTGAGTCATCGCTCAGGAG |
| Eno2-R | ATGTCCGGCAAAGCGAGCTTCATC |
| Bcat1-F | CAACTATGGAGAATGGTCCTAAGCT |
| Bcat1-R | TGTCCAGTCGCTCTCTTCTCTTC |
| Bcat2-F | CGCTCCTGTTCGTCATTCTCT |
| Bcat2-R | CCCACCTAACTTGTAGTTGCC |
| Gapdh-F | CCAGAACATCATCCCTGCCTCTACT |
| Gapdh-R | GGTTTTTCTAGACGGCAGGTCAGGT |
| Glut1-F | GGCCAAGAGTGTGCTAAAGAA |
| Glut1-R | ACAGCGTTGATGCCAGACAG |
| Gpi-F | TATTGTGTTCACCAAGCTCACACC |
| Gpi-R | TGGTAGAAGCGTCGTGAGAGGTC |
| Hk1-F | CACATGGAGTCCGAGGTTTATG |
| Hk1-R | CGTGAATCCCACAGGTAACTTC |
| Hk2-F | TGCCACCAGACTAAACTAGACG |
| Hk2-R | CCCGTGCCCACAATGAGAC |
| Ldha-F | TTGACCTACGTGGCTTGGAAG |
| Ldha-R | GGTAACGGAATCGGGCTGAAT |
| Pfkfb3-F | AGCCCGGATTACAAAGACTGC |
| Pfkfb3-R | GGTAGCTGGCTTCATAGCAAC |
| Pfkl-F | GTACCTGGCGCTGGTATCTG |
| Pfkl-R | CCTCTCACACATGAAGTTCTCC |
| Pfkm-F | AGCGTTTCGATGATGCTTCAG |
| Pfkm-R | GGAGTCGTCCTTCTCGTTCC |
| Pfkp-F | GACCTTCGTTCTGGAGGTGAT |
| Pfkp-R | CACGGTTCTCCGAGAGTTTG |
| Pgam1-F | CATCAGTAAGGATCGCAGGTATG |
| Pgam1-R | CTTCATTCCAGAAGGGCAGAG |
| Pgk1-F | GCCAAGTCGGTAGTCCTTATG |
| Pgk1-R | CCCAGCAGAGATTTGAGTTCTA |
| Pkm­­­­-F | AAGGGTGTGAACCTTCCTGG |
| Pkm-R | GCTCGACCCCAAACTTCAGA |

Table S2.

**Sequences of ChIP-qPCR primers**

| **Primer** | **Sequence (5’ to 3’)** |
| --- | --- |
| PFKP p1-F | ATAAGCCCGTGCTTCTATGG |
| PFKP p1-R | CAGCTCCACGTACTGAAATACT |
| PFKP p2-F | CCAGGCCAGTCCTGTTG |
| PFKP p2-R | GGGTGGAGGAAGGTATCG |
| LDHA p1-F | GCCTGTAATCCCAGCTCTTT |
| LDHA p1-R | ATGTTGGTCAGGCTGATCTC |
| LDHA p2-F | CTTGAGGCCAGGAGTTTGA |
| LDHA p2-R | CCTCCTGAGAAGATGGGATTAC |
